# Taxonomic and functional stability of sedimentary microbial communities in a pristine upwelling-influenced coastal lagoon

**DOI:** 10.1093/ismeco/ycaf241

**Published:** 2025-12-18

**Authors:** Jorge Rojas-Vargas, Guillermo Samperio-Ramos, Víctor F Camacho-Ibar, Silvia Pajares

**Affiliations:** Unidad Académica de Ecología y Biodiversidad Acuática, Institute of Marine Sciences and Limnology, National Autonomous University of Mexico (UNAM), Circuito exterior s/n, Ciudad Universitaria, Coyoacán, 04510, Mexico City, Mexico; Department of Biology, University of Western Ontario (UWO), 1151 Richmond St. N., London, Ontario, N6A 5B7, Canada; Department of Microbiology & Immunology, Schulich School of Medicine & Dentistry, University of Western Ontario (UWO), 1151 Richmond St. N., London, Ontario, N6A 5C1, Canada; Nutrient Cycling in Marine Ecosystems Research Group, Institute of Oceanological Research, Autonomous University of Baja California (UABC), Carretera Ensenada-Tijuana No. 3917, Fracc. Playitas, Ensenada, 22860, Baja California, Mexico; Nutrient Cycling in Marine Ecosystems Research Group, Institute of Oceanological Research, Autonomous University of Baja California (UABC), Carretera Ensenada-Tijuana No. 3917, Fracc. Playitas, Ensenada, 22860, Baja California, Mexico; Unidad Académica de Ecología y Biodiversidad Acuática, Institute of Marine Sciences and Limnology, National Autonomous University of Mexico (UNAM), Circuito exterior s/n, Ciudad Universitaria, Coyoacán, 04510, Mexico City, Mexico

**Keywords:** coastal sediments, seasonal upwelling, pristine environment, microbial communities, microbial diversity, functional potential, shotgun metagenomics

## Abstract

Coastal lagoons are dynamic transitional ecosystems shaped by complex hydrodynamic and biogeochemical processes. Their sediments host diverse microbial communities essential for nutrient cycling, organic matter sequestration, and pollutant degradation. However, the taxonomic and functional profiles of these communities remain poorly understood, especially in pristine systems. Here, shotgun metagenomics was used to investigate microbial diversity and functional potential in a seagrass-dominated coastal lagoon on the Mexican Pacific coast, influenced by seasonal upwelling and with minimal anthropogenic impact. Despite pronounced physicochemical gradients and oceanographic variability, these sediments harbored a diverse and taxonomically conserved microbial community. 60% of genera and 38% of species (with relative abundance >0.1%) were consistently shared across sites and the two upwelling seasons, with Gammaproteobacteria, Deltaproteobacteria, Alphaproteobacteria, Flavobacteria, and Actinobacteria as dominant taxa. Genes associated with nitrogen and sulfur metabolic pathways were consistently detected, suggesting the presence of a conserved functional core supporting key biogeochemical processes. In contrast, genes related to antibiotic resistance and virulence factors exhibited more heterogeneous distributions. Among measured physicochemical variables, only nitrate and ferric iron significantly influenced microbial community structure and its functional repertoire, suggesting that additional factors likely contribute to the broader distribution of these communities. These findings reveal a high degree of taxonomic and functional stability of microbial communities in a minimally impacted lagoon, providing a valuable baseline for understanding microbial dynamics in coastal sediments primarily shaped by oceanographic processes.

## Graphical Abstract

**Graphical Abstract:**
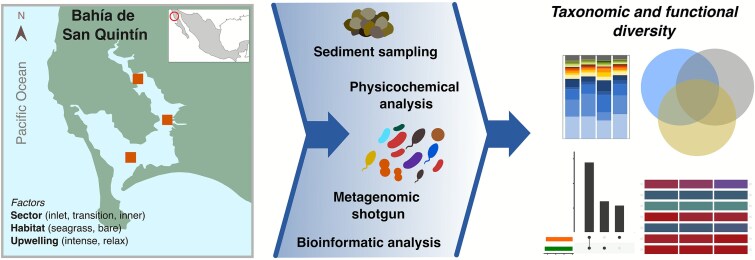

## Introduction

Coastal lagoons are dynamic ecosystems that function as crucial interfaces between marine and terrestrial environments. These shallow, semi-enclosed systems experience complex hydrodynamic processes, including tidal exchanges and terrestrial inputs, which influence nutrient availability and organic matter dynamics [1–4]. Sediments in these environments host highly diverse microbial communities that drive key biogeochemical processes, such as nitrogen and sulfur cycling, organic matter decomposition, and pollutant degradation [5, 6]. Despite their ecological relevance, microbial communities in lagoons primarily shaped by oceanic processes remain largely unexplored compared to those dominated by riverine inputs, limiting our understanding of their taxonomic diversity, functional roles, and responses to environmental changes.

Microbial diversity in coastal sediments is influenced by multiple factors, including sediment texture, physicochemical gradients, organic matter availability, macrophyte presence, and anthropogenic pressures, which collectively create distinct ecological niches [6–8]. Advances in 16S rRNA sequencing and metagenomics have greatly expanded our knowledge of microbial communities in anthropogenically impacted estuaries and coastal lagoons [6–10]. However, comparable investigations in relatively pristine lagoons remain scarce [11]. This gap is noteworthy because coastal systems are highly susceptible to environmental changes [1, 4, 12], and ocean–coast connectivity can strongly modulate microbial assemblages, metabolic capacities, and biogeochemical fluxes [13, 14]. Tidal forcing, marine nutrient pulses, and episodic oceanic intrusions modify salinity, redox conditions, and resource availability, producing sharp ecological gradients that can restructure microbial assemblages and their functions [13–15]. Therefore, understanding how these communities respond to environmental drivers is critical for assessing the resilience of coastal ecosystems under changing environmental conditions.

Bahía de San Quintín (BSQ) is a seagrass-dominated coastal lagoon on the northwest coast of the Baja California Peninsula, Mexico. Unlike many lagoons that receive significant freshwater inputs [3, 7, 16], BSQ experiences minimal anthropogenic disturbance. Instead, nutrient dynamics are primarily regulated by seasonal upwelling and tidal exchange with the Pacific Ocean [2], making it an ideal natural laboratory for studying microbial communities in relatively pristine coastal sediments subjected to oceanographic forcing. Previous studies in BSQ have identified aerobic denitrifying bacteria whose distributions are associated with sediment texture, pH, and organic matter content [17]. Nitrogen removal is predominantly mediated by denitrification and anaerobic ammonium oxidation (anammox), with additional contributions from noncanonical anammox processes, such as anaerobic ammonium oxidation coupled with the dissimilatory reduction of ferric iron [Fe(III), feammox] and Manganese (IV) [Mn (IV), manganammox] [18, 19]. Despite these insights, the overall taxonomic composition and functional potential of microbial communities in BSQ sediments remain largely unexplored, particularly under contrasting upwelling conditions. Metagenomic offers a powerful approach to fill this gap by simultaneously resolving microbial taxonomic identities and functional capabilities, including their potential responses to environmental stressors [20].

This study aims to assess the taxonomic diversity and functional capacity of sedimentary microbial communities in a highly productive, ocean-influenced coastal lagoon with minimal anthropogenic impact. The findings will enhance our understanding of microbial dynamics in coastal ecosystems influenced by eastern boundary upwelling systems and help elucidate the role of microbial communities in coastal sediments.

## Materials and methods

### Study area and sample collection

**Figure 1:**
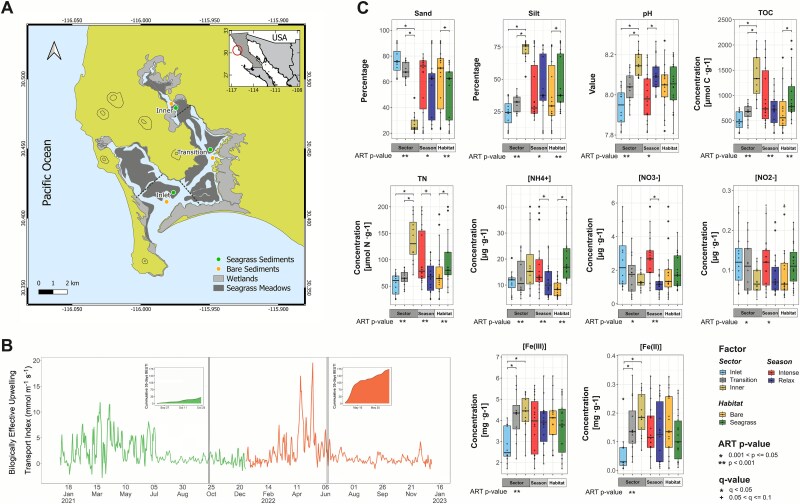
Environmental setting and sediment physicochemistry of the san Quintín coastal lagoon. Map of sampling sites across lagoon sectors (A). Daily biologically effective upwelling transport index (BEUTI) during the study period, including the cumulative BEUTI computed over a 30-day period (colored boxes) leading up to the fieldwork (indicated as vertical lines) under low BEUTI (green) and high BEUTI (orange) conditions (B). Boxplots of sediment physicochemical variables grouped by sector (inlet, transition, and inner), upwelling season (intense vs. relaxed), and habitat (bare vs. seagrass) (C). Boxes represent the inter-quartile range with the median indicated; whiskers extend to the most extreme value within 1.5 × IQR (n = 12 per sector; n = 18 per season and habitat). Asterisks beneath factor labels denote statistically significant main effects (*P* < .05) based on a three-factor ART ANOVA. Full statistical results are provided in Table S2.

BSQ is a shallow coastal lagoon (~2.5 m depth, 42 km^2^) with two distinct arms: Brazo San Quintín to the east, which remains almost unspoiled by human activity, and Bahía Falsa to the west, where moderate oyster aquaculture is practiced (Fig. 1A). The lagoon lies within a volcanic field of alkali-basaltic terrain, and its sediments are enriched with Fe and Mn oxide minerals [21]. Approximately 40% of the lagoon is covered by *Zostera marina* (eelgrass) meadows, which enhance organic matter availability in the sediments [22]. The region has a Mediterranean-type climate with semiarid conditions throughout the year [23]. Terrestrial inputs of water and nutrients are minimal, occurring only during exceptionally wet years. The southern boundary of the California Current System influences the ocean adjacent to BSQ. Lagoon hydrodynamics are primarily driven by tidal exchange with the Pacific Ocean, while nutrient inputs are dominated by seasonal coastal upwelling associated with the California Current, which peaks in spring and early summer [2, 24]. The combination of this nutrient regime with intense evaporation and low precipitation creates a hypersaline environment with an inverse salinity gradient that increases from the oceanic inlet (~34 g kg^−1^) to the inner sections (~38 g kg^−1^) [2, 25].

The Biologically Effective Upwelling Transport Index (BEUTI) was used to quantify upwelling intensity along the California Current System and estimate the nitrate (NO_3_^−^) content of upwelled waters [26], thereby characterizing the seasonal upwelling phenology in nearshore waters off BSQ. Daily BEUTI data (31° N) from 2021 to 2022 were obtained from [https://mjacox.com/upwelling-indices/]. Additionally, the cumulative monthly BEUTI values (the sum of daily values in the 30 days preceding each sampling) were considered for each sampling date (Fig. 1B).

Sediment samples were collected in two seasons based on the BEUTI index: October 2021 (upwelling relaxation) and June 2022 (intense upwelling). Sampling was conducted across three sectors of the Brazo San Quintín, based on previously reported physicochemical characteristics [2, 24]: (i) inlet, influenced by oceanic conditions, with an average seawater residence times of <2 days and the presence of sea lion rookeries; (ii) transition, with variable tidal intrusion and residence times of 5–7 days; and (iii) inner, with residence times >12 days. In each sector, sediments were collected at a vegetated site and at an adjacent bare site ~25 m from the eelgrass edge (Fig. 1A). Three independent surface sediment samples (0–5 cm) per site were collected by SCUBA at 1.5–2 m depth using Plexiglass cores and transported to the laboratory at 4°C within 4 h. Sediment samples were homogenized and separated into two subsamples under sterile conditions: one for physicochemical analysis and one preserved at −80°C for molecular analysis.

### Physicochemical characteristics of sediments

Sediment physicochemical analyses were performed according to [18]. Briefly, the granulometric composition was determined using a Horiba LA-910 particle-size analyzer. Porewater pH (NBS scale) was measured with a pH meter (OHAUS Starter 2100) after mixing fresh sediment with CO_2_-free artificial seawater at a 2:1 ratio (v:w). Total organic carbon (TOC) and total nitrogen (TN) were determined with an elemental analyzer (Vario Isotope Select). After extracting exchangeable nutrients with 2 M KCl, ammonium (NH_4_^+^) was measured by the optimized indophenol method [27], while NO_3_^−^ and nitrite (NO_2_^−^) were analyzed with an AA3-HR segmented-flow autoanalyzer (SEAL Analytical Ltd) [28]. The mobilizable fractions of ferrous iron [Fe(II)] and total Fe were extracted with 0.5 M HCl and 0.25 M hydroxylamine-HCl, respectively. Fe(II) was quantified with a FLAME-S UV–Vis spectrophotometer (Ocean Insight) following a modified ferrozine-based colorimetric method [29]. The amount of Fe(III) was estimated as the difference between total extractable Fe and Fe(II).

﻿ As the physicochemical data did not meet normality assumptions, an aligned-rank-transform (ART) ANOVA was applied using the “ARTool” package v0.11.2 [30] in R v4.4.1 [31] to test main and interaction effects of sector, season, and habitat. When a multilevel factor showed a significant effect (*P* < .05), post hoc comparisons were performed using a false discovery rate (FDR)–adjusted Dunn test for sector with the “FSA” package v0.9.5 [32], or Wilcoxon pairwise tests for season and habitat with the “stats” package in R.

### DNA sequencing, metagenomics assembly and taxonomic assignment

Metagenomic DNA was extracted using the DNeasy PowerSoil Pro Kit (Qiagen, Germany). DNA extracts from the three replicates per site were pooled in equal volumes to obtain a representative composite sample and reduce within-site variability prior to sequencing. Pooled samples were sent to Microomics (Barcelona, Spain) for shotgun metagenomic sequencing on an Illumina NovaSeq 6000 platform (2x150 bp paired-end reads), following Microomics’ protocol (http://www.microomics.com).

Raw reads, averaging 53.4 ± 10.2 million paired reads per sample, were processed using Trimmomatic v0.39 [33] to remove adapters and retain sequences with Q > 20. After quality control, 59.5%–82.8% of paired reads per sample were retained (Table S1). Trimmed reads were assembled with MEGAHIT v1.1.4 [34] using the parameters —k-min 27 —k-max 127 —k-step 10 —kmin-1pass, yielding between 402 223 and 970 992 contigs, with an average assembly size of 376 ± 119 Mbp (Table S1).

Taxonomic classification was performed with Kaiju v1.8.0 [35] using default parameters and the NCBI BLAST nr + euk database. Outputs were transformed with Kraken2 v2.1.3 [36] and count tables were visualized with the “pavian” package v1.2.1 [37] in R. The relative abundances (RAs) of prokaryotic taxa were calculated after removing the eukaryotic and viral fractions and visualized in class-level barplots using the “ggplot2” package v3.5.1 [38] and genus- and species-level heatmaps using the “ampvis2” package v2.8.9 [39] in R. Differentially abundant genera across factors were identified with the “ANCOM-BC” package v2.6.0 [40], applying the FDR method to calculate q-values and the struc_zero = FALSE option. Genera with q < 0.05 were considered significant. Taxa with RA >0.1% were selected in R to identify unique and shared genera and species across conditions.

### Diversity analyses and functional annotation

Taxonomic diversity was estimated from metagenomic taxonomic units assigned by Kaiju at the genus and species levels. Alpha diversity indices (observed richness, Shannon) were calculated with the “phyloseq” package v1.48.0 [41] in R. Alpha diversity comparisons among sectors (inlet, transition, inner) were performed with Kruskal-Wallis test using “FSA,” whereas habitat (bare vs. seagrass) and upwelling season (intense vs. relaxed) were compared with Wilcoxon rank-sum test in R. For beta diversity, taxonomic count tables were centered log-ratio (CLR) transformed to calculate Aitchison distances [42] with the “microbiome” package v1.26.0 [43]. These distances were used to perform Principal Component Analysis (PCA) and dispersion analysis with the “vegan” package v2.6–8 [44]. Differences in beta diversity were tested with permutational analysis of variance (PERMANOVA).

Associations between microbial community structure and environmental variables were evaluated by redundancy analysis (RDA) using “vegan.” Genus-level abundances were Hellinger-transformed, and environmental variables were standardized prior to RDA [45]. Predictors with Pearson correlations |r| ≥ 0.7 were first discarded, and variables with variance inflation factors (VIFs) > 10 were excluded. Stepwise forward-backward selection with the ordistep function was applied with the remaining variables to identify the significant predictors, whose contributions were evaluated using a permutation test. The RDA biplot was generated with base R plotting functions.

Coding sequences (CDSs) were predicted from the metagenomic assemblies using Prodigal v2.6.3 [46], and functional annotation was performed with DIAMOND blastp v2.1.8 [47] applying ≥40% sequence identity, ≥60% coverage, and E-value <1 × 10^−10^. Although homology-based inference is possible at ~30% identity [48], the inclusion of a 60% coverage and a stringent E-value reduce false positives and ensures robust matches. The NCyc and SCyc databases were used to predict genes involved in nitrogen and sulfur metabolism pathways [49, 50]; BacMet v2.0 [51] for metal-associated genes; CARD v3.0.7 [52] for antibiotic resistance genes (ARGs); and VFDB v2.3 [53] for putative virulence factors.

Pathway completeness for nitrogen and sulfur metabolism was calculated as the proportion of pathway reactions represented by at least one predicted gene. Mean completeness values and the corresponding percentages of gene families averaged across samples sharing a given factor level (sector, habitat, or season) were visualized on heatmaps with “ggplot2.” Significant differences in gene proportions and pathway completeness were evaluated with Kruskal–Wallis, followed by the FDR-adjusted Dunn’s *post hoc* test for the sector factor or Wilcoxon tests for habitat and season comparisons, as previously described. The RA of predicted genes was calculated as the percentage of CDSs in each metagenome. Shared and unique protein-encoding genes across factors were visualized using UpSet diagrams with the “UpSetR” package v1.4.0 [54]. Relationships between predicted functional genes and environmental variables were assessed using RDA as described above.

## Results and discussion

### Physicochemical characteristics of Bahía de San Quintín sediments

Spatial and seasonal patterns were evident across the 10 physicochemical variables measured. All variables differed significantly among sectors, eight between upwelling seasons, and five between habitats (*P* < .05; Fig. 1C and Table S2). Several variables also showed significant sector × season and sector × habitat interactions, indicating that the magnitude of sector differences shifts with changes in upwelling intensity and vegetation cover.

﻿Granulometry followed a pronounced inlet-to-inner gradient. Sand content decreased significantly from 76.2% ± 10.8% at the inlet to 27.4% ± 8.0% at the inner, accompanied by a corresponding increase in silt content, which reduces permeability and oxygen availability, favoring anoxic conditions in this sector [18]. A strong significant sector × season × habitat interaction (*P <* .001) further indicated that seasonal and habitat effects on grain size varied by sector: the sandiest sediments occurred in the bare inlet sector during intense upwelling, whereas the siltiest occurred in inner seagrass beds during the relaxed season (Table S2).

Sediment chemistry showed similar significant trends (*P* < .05; Table S2). For instance, pH increased from inlet to inner (7.94 ± 0.09 to 8.15 ± 0.06) and was higher during the relaxed season (8.10 ± 0.08) compared to the intense upwelling period (7.99 ± 0.12). TOC and TN increased ~2.7-fold toward the inner sector, reflecting organic matter accumulation in areas with reduced hydrodynamic renewal [2], and were higher in seagrass habitats than in bare sediments (TOC: 989.4 ± 483.6 vs 704.6 ± 442.8 μmol C·g^−1^; TN: 96.4 ± 45.5 vs 75.2 ± 46.8 μmol N·g^−1^), suggesting enhanced carbon and nitrogen retention in vegetated sediments. NH₄^+^ concentrations were higher in the inner sector (18.53 ± 12.2 μg·g^−1^), in seagrass beds (19.98 ± 9.27 μg·g^−1^), and during intense upwelling (17.07 ± 10.47 μg·g^−1^). In contrast, NO_3_^−^ and NO_2_^−^ declined from inlet to inner (NO_3_^−^: 2.58 ± 1.68 to 1.42 ± 0.59 μg·g^−1^; NO_2_^−^: 0.13 ± 0.07 to 0.07 ± 0.03 μg·g^−1^), but increased during intense upwelling (NO_3_^−^: 1.13 ± 0.48 vs 2.68 ± 1.38 μg·g^−1^; NO_2_^−^: 0.09 ± 0.05 vs 0.12 ± 0.06 μg·g^−1^), particularly in the inlet sector. Both Fe(III) and Fe(II) increased toward the interior (Fe(III): 2.81 ± 0.83 to 4.33 ± 0.89 mg·g^−1^; Fe(II): 0.06 ± 0.07 to 0.20 ± 0.08 mg·g^−1^), with no significant seasonal or habitat differences. Fe(III) serves as an electron acceptor for ammonium oxidation under anoxic conditions, contributing to nitrogen loss via noncanonical anammox in BSQ sediments, with the highest potential rates observed in the transition and inner sectors [18].

### Distribution of the microbial taxonomic composition in Bahía de San Quintín sediments

**Figure 2:**
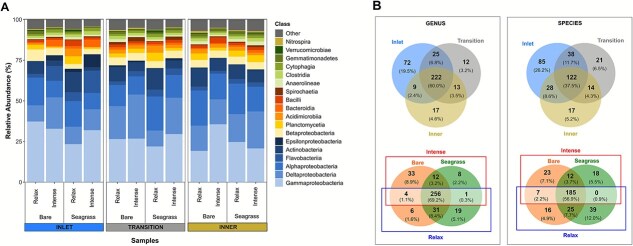
Taxonomic profiles of prokaryotic communities across the san Quintin coastal lagoon. Comparison of the relative abundances (RA) at the class level, showing only classes present in at least one sample with RA ≥1% (A). Number and percentage of shared and unique taxa with RA > 0.1% at the genus and species levels (B). The distribution of ﻿prokaryotic taxa is shown for the three analyzed factors: Sector, habitat, and upwelling season.

Contigs (~8.1 million) were predominantly bacterial (98.2%), with minor fractions of Eukaryotes (0.9%), Archaea (0.8%), and Viruses (0.1%). The prokaryotic component comprised 93 classes and 2237 genera, with Gammaproteobacteria (mean RA 27.4%), Deltaproteobacteria (16.8%), Alphaproteobacteria (15.2%), Actinobacteria (7.8%), and Flavobacteria (4.5%) as the dominant groups (Fig. 2A). These taxa are commonly found in coastal sediments and play key roles in biogeochemical cycles [6, 7, 10, 14].

The most abundant bacterial genera were *Vibrio*, *Woeseia*, and *Psychrobacter* (Fig. S1A, S2A). *Vibrio* dominated the inlet sector during intense upwelling (mean RA 11.2%) and in seagrass habitats (7.8%), with *V. splendidus* as the most abundant species (1.62%; data not shown). This species thrives in nutrient-rich marine environments, with potential implications for aquaculture and human health [55]. Its high abundance may be influenced by oyster farming in Bahia Falsa—the lagoon arm adjacent to this sector—and the presence of sea lions at the BSQ inlet. *Woeseia*, a ubiquitous marine genus ﻿with versatile metabolic capabilities [56, 57], had a mean RA of 2.47%, with *W. oceani* as the most prevalent species (2.02%; Fig. S1B, S2B). This species may contribute to organic carbon remineralization and produce the greenhouse gas nitrous oxide (N_2_O) via denitrification [58, 59]. *Psychrobacter* (2.34%) exhibited a heterogeneous distribution, consistent with previous observations in BSQ [17]. Aerobic denitrifying *Psychrobacter* strains, particularly *P. piscatorii*, *P. submarinus* and *P. pacificensis,* have been isolated from BSQ sediments [17]. This study confirmed the presence of these species, along with 47 additional *Psychrobacter* taxa, of which 66% remain undescribed (data not shown).

Other abundant coastal sediment genera included *Ilumatobacter* (2.3%), a hydrocarbon degrader with ammonia transporters [60], and *Streptomyces* (1.6%), known for producing secondary metabolites such as antibiotics [61, 62]; both were most abundant in the transition sector during relaxed season (Fig. S1A). *Halioglobus* (1.4%), with nitrogen-removal potential [63], also peaked in this sector. *Methyloceanibacter* (1.3%), capable of methane oxidation and N_2_O production [64], was most abundant in the inner sector during relaxation, whereas *Pseudomonas* (1.17%) was evenly distributed and might contribute to ammonification and denitrification in these sediments [65, 66]. Sulfur-cycling genera included *Sulfurovum* (1.37%), a sulfur oxidizer that couples sulfur oxidation to the reduction of Fe oxides and NO_3_^−^ [67, 68], and the sulfate reducers *Desulfosarcina* (0.67%) and *Desulfovibrio* (0.54%) (Fig. S1A, S2A), previously linked to manganammox in BSQ sediments [19]. Collectively, these taxa likely contribute to organic matter transformation and nutrient cycling in the lagoon.

Archaea were also present in BSQ sediments (Table S3), with Halobacteria (0.08%–0.29%) as the most abundant class, particularly in the inlet sector, followed by Methanomicrobia (0.14%–0.26%), which showed a homogeneous distribution. Halobacteria thrive in saline, low-oxygen environments using nitrogen or sulfur compounds as electron acceptors [69, 70], whereas Methanomicrobia are widespread methanogens frequently found in estuarine sediments that may also contribute to Fe(III) reduction [70, 71] in BSQ. The dominant archaeal genera were *Nitrosopumilus* (0.001%–1.15%, within Nitrososphaeria class), *Methanosarcina* (0.05%–0.10%), and *Methanoculleus* (0.01%–0.04%) (data not shown). Archaeal RA in BSQ (0.64%–1.70%) was comparable to values from Sundarbans mangrove sediments in the Bay of Bengal (1%–3%) [72], but lower than those reported for the Venice lagoon (2.3%–4.7%) [73], the Pearl River estuary (10%–42%) [74], and the northwestern coast of Baja California (2.4%–12%) [75]. The low archaeal RA in BSQ and the Sundarbans may reflect limited anthropogenic influence, although this hypothesis requires further evaluation to better understand the potential influence of human activity on archaeal abundance in sedimentary environments.

A highly conserved core microbiome was identified in BSQ. Remarkably, 60% of the most abundant genera (RA >0.1%; 370 out of 2237 genera) and 38% of the most abundant species (RA >0.1%; 325 out of 18 458) were shared among the three sectors (Fig. 2B). Across habitats and seasons, 69% of the dominant genera and 57% of the dominant species were consistently shared, indicating a high degree of microbial stability despite periodic environmental fluctuations [76]. This apparent stability may reflect both the ecological persistence of groups well adapted to lagoon physicochemical variability, through functional redundancy and metabolic versatility [77, 78], and methodological factors, as conservative metagenomic assembly and filtering preferentially recover abundant taxa over rare or transient ones [79].

Although methodological differences limit direct comparisons—this study employed metagenomics at the genus and species levels, whereas most previous studies relied on amplicon sequencing—, the core microbiome in BSQ is notably high. For instance, a 15-month study in sediments from the relatively pristine Havre-aux-Maisons lagoon (Canada) found that at most 20% of operational taxonomic units (OTUs) were shared across samples [11]. In contrast, in the more disturbed Mar Menor lagoon (Spain), only nine OTUs with RA >0.1% formed the core microbiome, representing 2.4%–13.8% of total 16S rRNA sequences and exhibiting pronounced spatial heterogeneity [8]. Other lagoons have shown high microbial stability despite environmental variability. For example, bacterial communities in surface waters of the Patos Lagoon estuarine system (Brazil) remained stable despite strong salinity and nutrient gradients [78], indicating that functional redundancy and dispersal processes can sustain compositional stability under fluctuating conditions.

Among the 370 genera with RA >0.1%, only a small subset exhibited significant abundance differences (q < 0.05): 18 by sector, 7 by habitat, and 12 by upwelling season, highlighting the overall compositional stability of these sediments under varying conditions (Figs S3A–C). Most sector-level differences were concentrated at the lagoon entrance: 15 genera differed between the inlet and inner sectors, 9 between inlet and transition, and 8 between transition and inner, whereas the inner sector differed from the inlet by only three genera and from the transition by just one (Fig. S3A). This inlet–inner contrast likely reflects the influence of tidal marine intrusions, which introduce ocean-derived taxa, nutrients, and labile organic substrates into the lagoon [24, 80]. Inlet-enriched genera such as *Sulfurovum, Flaviramulus, Arenitalea,* and *Algibacter*, key players in sulfur cycling and organic matter decomposition in marine sediments [67, 68], may be stimulated by pulses of phytoplankton-derived dissolved organic matter and nutrients entering from the ocean [81]. Such patterns align with observations that periods of increased marine connectivity induce shifts in dissolved organic matter properties and microbial community structure in coastal lagoons [15], further supporting the role of lagoon–ocean exchanges in shaping microbial assemblages. Conversely, *Hyphomicrobium, Filomicrobium,* and *Methyloceanibacter* were significantly more abundant in the inner sector, possibly influenced by higher levels of ammonia, TOC, TN, and Fe. Elevated ammonia concentrations in this area also support a higher potential for noncanonical anammox [18].

Seven genera with RA >0.1% were significantly more abundant in seagrass sediments than in bare habitats (q < 0.05, Fig. S3B). Among them, *Tenacibaculum*, *Polaribacter*, *Mangrovimonas*, and *Lutibacter* are known to degrade complex organic matter [82–84]. Vegetated sediments provide a more dynamic microbial environment through root-driven oxygen release and inputs of organic exudates and plant detritus, enhancing microbial activity and nutrient recycling compared to bare sediments [85, 86]. During intense upwelling, 11 genera were significantly more abundant than during relaxed upwelling (q < 0.05, Fig. S3C), possibly due to increased nutrient inputs that stimulate microbial growth. In contrast, Ca. *Microthrix* was the only abundant genus significantly enriched during relaxed upwelling, likely due to higher organic carbon accumulation and reduced hydrodynamic renewal, which may favor filamentous bacteria [87].

### Community diversity distribution in Bahía de San Quintín sediments

**Figure 3:**
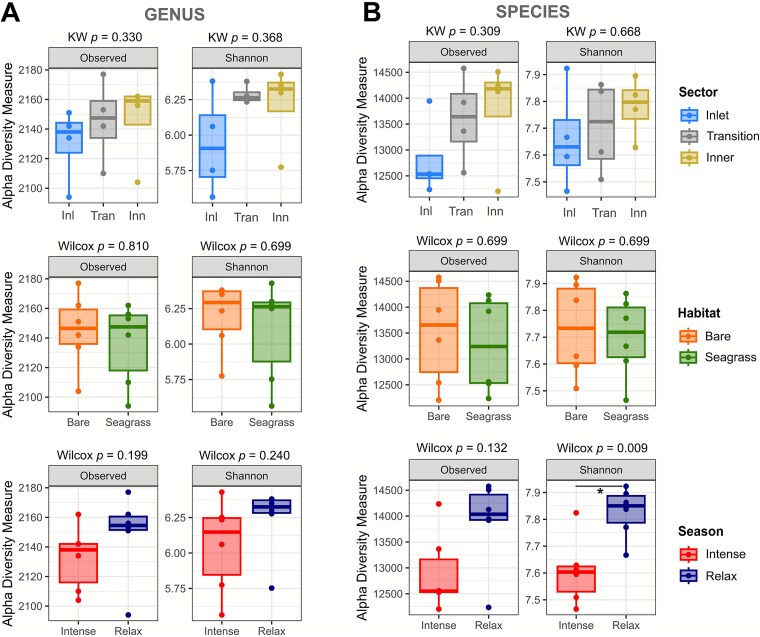
Boxplot comparisons of prokaryotic alpha diversity indices at the genus (A) and species (B) level, across sectors, habitats, and upwelling seasons. Statistical differences were evaluated using the Kruskal–Wallis (KW) test for sectors and the Wilcoxon rank-sum (Wilcox) test for habitats and seasons (n = 4 per sector; n = 6 per season and habitat). Asterisk denotes statistically significant effects (*P* < .05).

Alpha diversity in BSQ sediments falls within the range reported for other metagenomic studies of coastal and marine sediments [88, 89]. At the genus level, observed richness ranged from 2094 to 2177, with Shannon index values of 5.56–6.75 (Fig. 3A). At the species level, observed richness ranged from 12 204 to 14 576, with Shannon values of 7.47–7.92 (Fig. 3B). Species-level diversity in BSQ was lower than in mangrove sediments from Baía de Todos os Santos, Brazil (Shannon 10.6–11.2) [89], which contain 1%–6% TOC [90], but higher than in Persian Gulf coastal sediments (Shannon 3.83–4.01) [88]. The elevated diversity in mangrove sediments is consistent with their higher TOC and continuous input of root exudates and litter, whereas BSQ sediments contain ~0.6% TOC [21] and lack mangrove root networks, limiting energy availability.

Overall, alpha diversity did not differ significantly across sectors or habitats (Fig. 3A and B). At the species level, however, Shannon diversity was significantly higher during relaxed upwelling than during intense upwelling (Wilcoxon *P* = 0.009), whereas observed richness remained unchanged (*P* = 0.132). Prokaryotic diversity also increased gradually from the inlet toward the inner sector, likely reflecting higher organic matter concentrations and longer water residence times in the lagoon interior. In general, BSQ sediments showed broadly stable microbial diversity, with only modest seasonal shifts associated with upwelling intensity.

**Figure 4:**
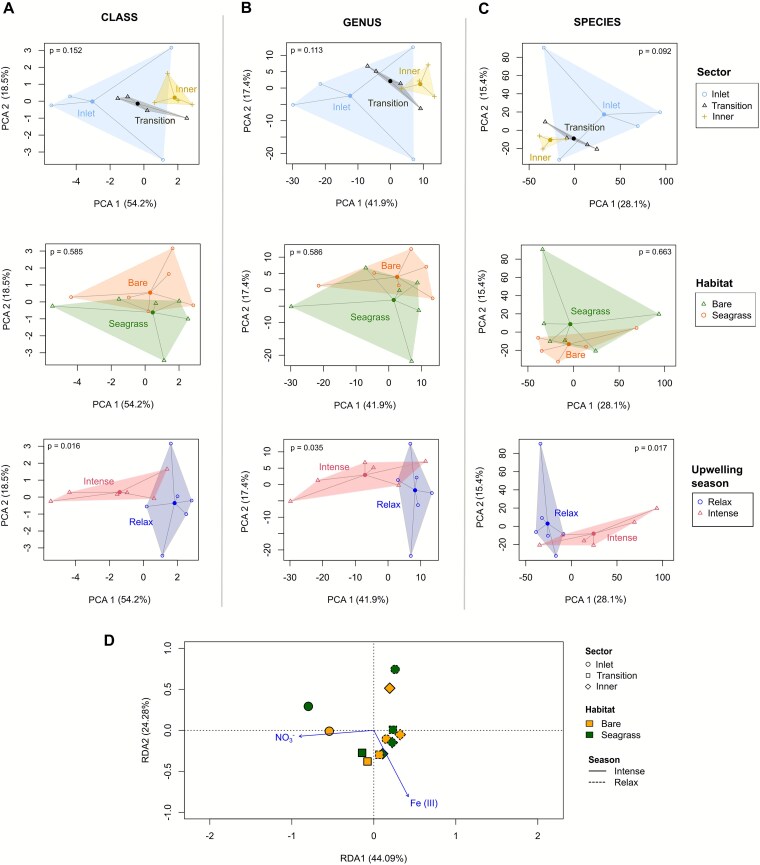
Beta diversity and redundancy analysis (RDA). Principal component analysis (PCA) and dispersion analysis of prokaryotic communities by sectors, habitats, and upwelling season at class (A), genus (B), and species (C) levels. PERMANOVA *P*-values are shown in each plot. RDA plot depicting the relationship between the sedimentary communities at the genus level and the physicochemical factors (D). Length and angle of arrows: Extent of correlation between significant environmental factors (*P* < .05, 999 permutations) and RDA axes.

Although coastal lagoon sediments often show marked microbial heterogeneity and turnover [7, 8, 11], beta-diversity analysis in BSQ revealed no significant differences in community dispersion among sectors or habitats (Figs 4A–C). Upwelling season was the only factor associated with compositional shifts: communities differed significantly between relaxed and intense upwelling at the class, genus, and species levels (Figs 4A–C, PERMANOVA, *P* < .05). Even so, dispersion tended to be higher in the inlet sector and in vegetated sediments. The inlet experiences shorter water-residence times and stronger, tidally driven exchanges with the open ocean, exposing its communities to greater physicochemical variability. Vegetated sediments, in turn, receive organic inputs that create diverse microhabitats and may broaden compositional spread. Together, upwelling emerged as the principal driver of community change in BSQ, with hydrodynamics and benthic habitat exerting subtler influences.

The RDA analysis revealed no clear clustering patterns of sedimentary communities by sector, habitat, or season, with the first two axes accounting for 66.5% of the variance (Fig. 4D). Among the environmental assessed variables, only two significantly influenced community structure: NO_3_^−^, associated with oceanic influence, and Fe(III), indicative of iron-rich sediments (Table S4). Together, these variables explained 39.3% (adj. R^2^) of the ﻿variance in genus-level abundances (Table S5), suggesting that microbial structure in BSQ sediments is largely modulated by redox conditions and ocean-derived nutrient gradients. Other environmental or biotic factors not included in the model may also shape community structure. For instance, TOC and TN showed strong spatial gradients but were highly correlated with sediment texture and pH (|r| > 0.7; Table S4), limiting their independent explanatory power.

### Predictive functional analysis of microbial communities in Bahía de San Quintín sediments

**Figure 5:**
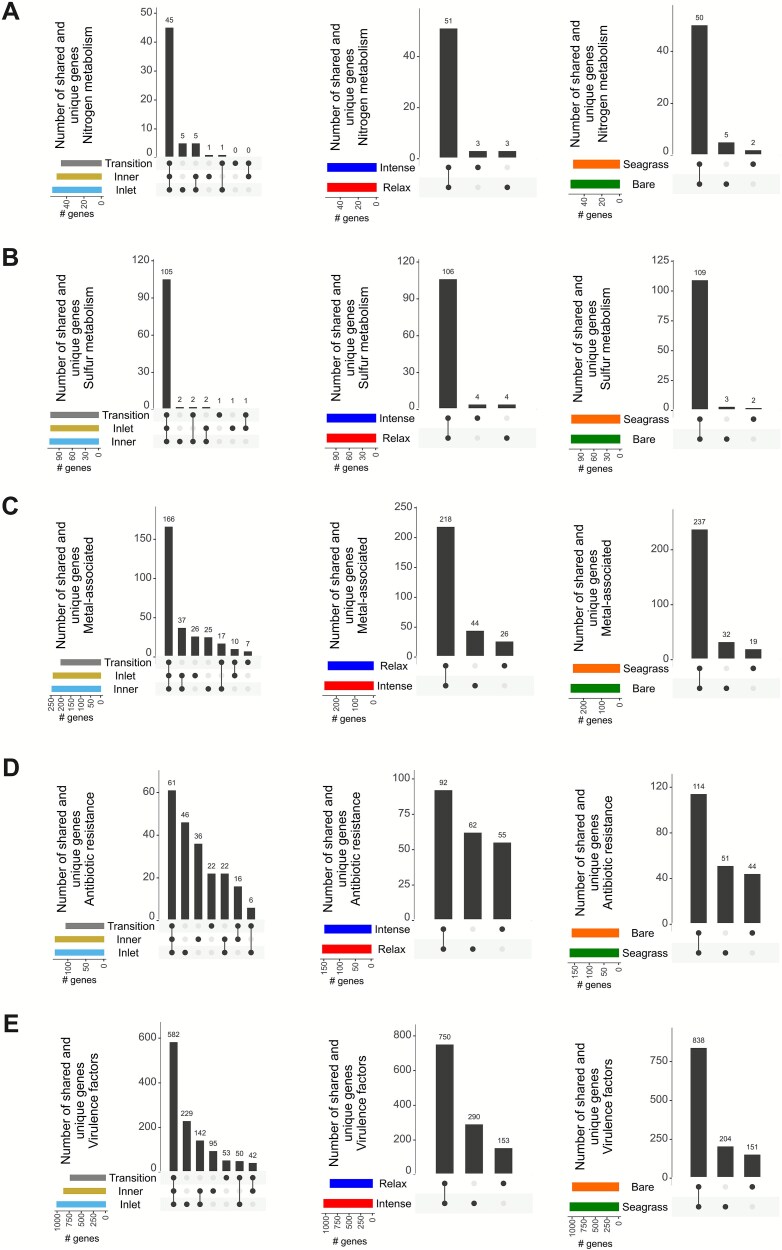
UpSet diagrams showing shared and unique protein-encoding genes related to different functional categories based on presence/absence comparisons. Panels represent genes involved in: Nitrogen metabolism (A), sulfur metabolism (B), metal-associated genes (C), antibiotic resistance genes (D), and virulence factors (E) for the three analyzed factors (sector, upwelling season, and habitat). The upper bar plot indicates the number of genes present in each intersection of factor levels. The matrix of connected dots shows which combinations of factor levels are involved in each intersection. The left bar plot displays the total number of genes detected per individual factor level.

Of the 10.2 million CDSs in the BSQ metagenomes, 167 321 (1.6%) were assigned to one of the five functional categories analyzed, representing 1861 gene families (Figs 5A–E). These included nitrogen-cycle (57 of the 79 genes catalogued in the reference database; 34 701 CDSs), sulfur-cycle (114/207; 63 823 CDSs), metal-associated (288/595; 16 044 CDSs), ARG (209/4805; 3701 CDSs), and virulence-factor (1193/7818; 49 052 CDSs) gene families. Predicted virulence-factor and ARG gene families were relatively lower (15.3 and 4.3%, respectively) than those for nitrogen- and sulfur- cycles (72.2 and 54.1%, respectively).

Compared with more disturbed coastal systems, BSQ harbored a broader repertoire of nitrogen- and sulfur-cycle genes but a much smaller resistome and virulome, consistent with its limited anthropogenic influence. For example, the Pearl River estuary (China) contained only 19 nitrogen-cycle genes [91]; Qi’ao mangrove sediments (China) held 19 nitrogen- and 18 sulfur-cycle genes but 270 ARGs [92, 93]; the Khambhat Gulf (India) had >2300 ARGs [94]; the Persian Gulf (Kuwait) hosted 609 ARGs [88]; and Baltic Sea sediments harbored 1653 virulence-factors and 563 metal-resistance genes [95]. The comparatively low abundance of ARGs and virulence factors in BSQ may reflect background levels typical of minimally disturbed environments. Such baselines serve as valuable references for future assessments, as increasing ARGs in natural microbiomes is considered as an early-warning indicator of emerging anthropogenic pressures [96, 97].

**Figure 6:**
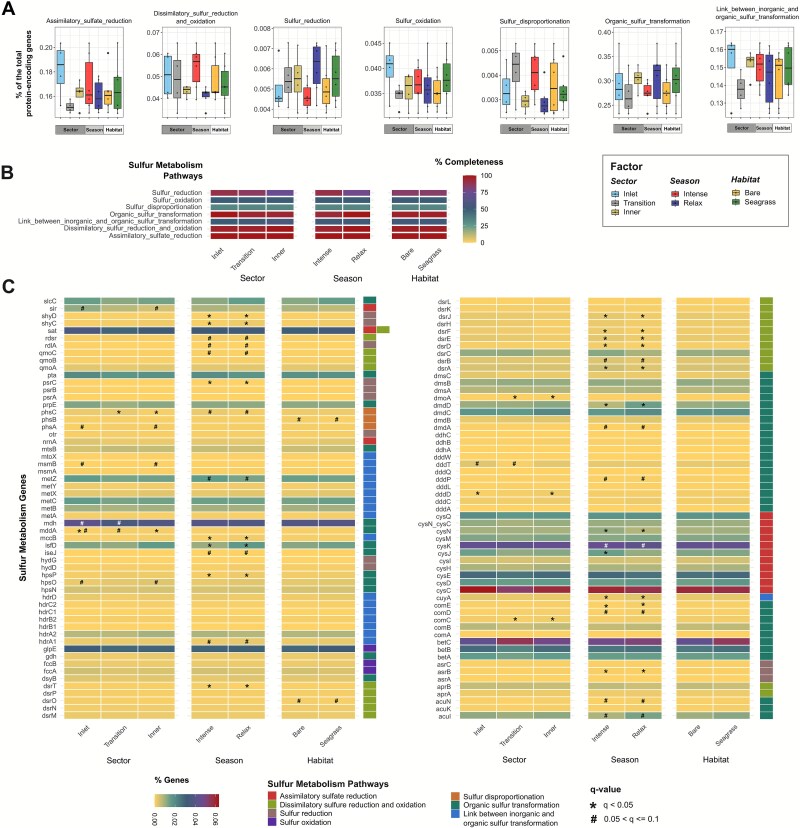
Sulfur-cycling gene repertoire in BSQ sediments. Relative abundance (% of CDSs) of sulfur-cycling gene families within each metabolism pathway, averaged across factor levels for lagoon sector, upwelling season, and habitat (A). Heatmap of mean completeness (%) of sulfur metabolism pathways for samples grouped by factor level (sector, season, or habitat) (B). Percentages of sulfur-metabolic gene families detected within each factor (C). Significant differences were found only in panel C. Asterisks (^*^) denote significant differences (q < 0.05), and crosshatches (#) indicate marginal significance (0.05 < q < 0.10) based on FDR-adjusted Dunn tests for sectors and Wilcoxon tests for seasons and habitats.

**Figure 7:**
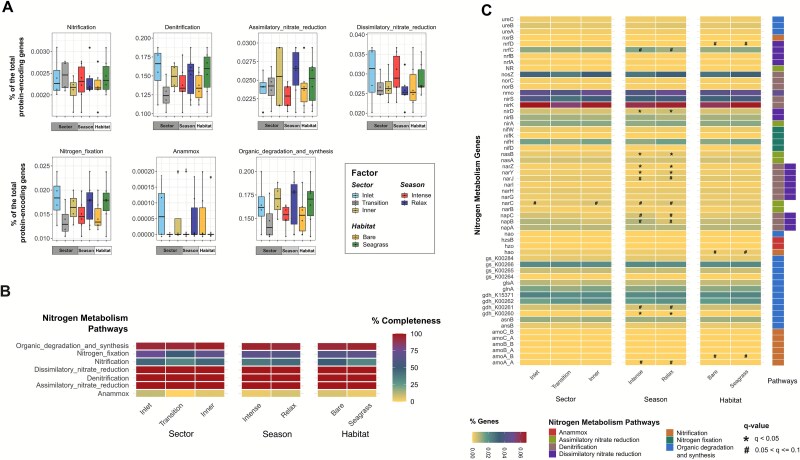
Nitrogen-cycling gene repertoire in BSQ sediments. Relative abundance (% of CDSs) of nitrogen-cycling gene families within each metabolism pathway, averaged across factor levels for lagoon sector, upwelling season, and habitat (A). Heatmap of mean completeness (%) of nitrogen pathways for samples grouped by factor level (sector, season, or habitat) (B). Percentages of nitrogen-metabolic gene families detected within each factor (C). Significant differences were found only in panel C. Significance symbols are as in Fig. 6.

Sulfur-metabolism genes were the most abundant across categories, particularly those involved in organic-sulfur transformations (0.29% ± 0.09%), assimilatory sulfate reduction (0.17% ± 0.04%), and inorganic/organic sulfur transformations (0.15% ± 0.02%) (Fig. 6A). Nitrogen-cycle genes were also well represented, notably those related to denitrification (0.14% ± 0.04%) and organic-nitrogen metabolism (0.16% ± 0.04%) (Fig. 7A). In contrast, metal-associated genes, ARGs, and virulence factors showed the lowest RA (0.01% ± 0.04%, 0.05% ± 0.15%, and 0.03% ± 0.12%, respectively) (Figs S4–S6).

A large fraction of nitrogen-related (79–89%; 45–51/57) and sulfur-related genes (92–96%; 105–109/114) was consistently shared across sectors, seasons, and habitats (Fig. 5A and B), indicating a stable functional core involved in biogeochemical cycling. This high and widespread metabolic potential, along with a highly conserved taxonomic core, suggests that BSQ communities can sustain key biochemical processes despite environmental fluctuations [77, 98]. In contrast, metal-associated genes showed moderate overlap across factors (58%–82%), whereas ARGs (29%–55%) and virulence factors (49%–70%) varied more widely (Figs 5C–E), suggesting that local selective pressures might influence the distribution of these traits [97, 99]. Additionally, the distribution of functional gene families was spatially and seasonally uniform (Figs 6A, 7A, S4–S6), with only four families differing significantly in RA (q < 0.05): chromium and selenium metabolism genes (Fig. S4), streptogramin-A resistance genes (Fig. S5), and adherence-related virulence factors (Fig. S6), all enriched in the inlet sector.

Key nitrogen and sulfur cycling pathways were mostly complete across sectors, seasons, and habitats (Fig. 6B, 7B). Denitrification, assimilatory and dissimilatory NO_3_^−^reduction, and assimilatory sulfate reduction each exhibited 100% pathway completeness across all factors. Organic nitrogen degradation and synthesis were nearly complete (93.8%–96.9%), as well as dissimilatory sulfur reduction/oxidation (95.2%–100%, reaching full completeness in the inner sector and during relaxed upwelling), organic sulfur transformation (93.3%–100%, fully complete in the inlet sector and during relaxed upwelling), and sulfur reduction (75%–90%, with the highest completeness in the inlet and during intense upwelling). In contrast, anammox (up to 10% of completeness at the inlet), nitrification (up to 50% in bare sediments), nitrogen fixation (up to 75% at the inlet), sulfur disproportionation (33.3% across all levels), sulfur oxidation (50% in all levels), and inorganic/organic sulfur transformation (50%–56.4%) showed lower completeness and no significant differences within and among factors. Overall, patterns were consistent across sites and seasons, indicating a broadly uniform biogeochemical potential in BSQ sediments.

Notably, nitrification was incomplete, mainly in the transition sector and seagrass habitats (33.3%), because the *amoA* and *amoC* genes were not detected (Fig. 7B and C). This may reflect limitations in gene prediction thresholds, database gaps, or the presence of uncharacterized or misannotated *amo* genes [49]. Indeed, *amoA* has been detected previously in BSQ sediments using qPCR (unpublished data). Similarly, the anammox pathway was represented only by *hzsB* and *hzo*, while key genes (*hzsAC, hdh*) were undetected (Fig. 7C), again suggesting detection or annotation limitations rather than true gene absence.

Gene-level analysis revealed that upwelling seasons significantly influence the RA of a few nitrogen and sulfur genes (q < 0.05; Fig. 6C, 7C). During intense upwelling, RA increased for genes involved in denitrification (*narZ*), assimilatory NO_3_^−^ reduction (*nasB*), and dissimilatory sulfate reduction and sulfur oxidation (*dsrA, dsrD, dsrE, dsrF, dsrJ, dsrT*), likely in response to inputs of nutrient-rich waters (Fig. 1B). At the sector level, the inlet exhibited significantly higher RA of genes for organic sulfur transformation (*dddD, mddA*), streptogramin/streptogramin-A resistance, selenium and chromium metabolism, and adherence factor (Figs 6C, S7A–C). No significant differences were observed across habitats (Figs 6C, 7C, S7A–C). These results suggest that, despite seasonal upwelling and spatial physicochemical gradients, a stable functional core sustains the metabolic capacity of BSQ sediments.

The RDA of predicted functional gene counts showed no clear clustering by sector, habitat, or season, with the first two axes explaining 45.1% of the variance (Fig. S8). Consistent with the taxonomic analyses, NO₃^−^ and Fe(III) were the only variables significantly influencing the functional gene profiles, together accounting for 21.4% of the variance (Tables S6, S7). This suggest that additional environmental or biotic drivers likely contribute to the broader distribution of functional genes in BSQ sediments.

## Conclusions

Despite pronounced physicochemical gradients and seasonal upwelling, BSQ sediments harbored a diverse and remarkably stable microbial community. A substantial core microbiome within the most abundant taxa (RA >0.1%), comprising 60% of genera and 38% of species, was consistently shared across sectors, habitats, and seasons. This taxonomic core was mirrored by a conserved set of nitrogen- and sulfur-cycling genes with nearly complete pathways. Together, these features indicate a microbial assemblage well adapted to fluctuating yet nutrient-rich conditions, likely favored by the lagoon’s low anthropogenic impact. In contrast, ARGs and virulence genes exhibited lower RA and higher heterogeneity, pointing to localized selective pressures. Overall, these findings underscore the adaptability and functional robustness of BSQ sediment microbiomes. Establishing such baselines in relatively undisturbed coastal systems is crucial for predicting microbial responses to environmental changes and identifying early signs of ecosystem disruption.

## Supplementary Material

Supplemental_figures_ycaf241

Supplemental_tables_ycaf241

## Data Availability

Shotgun raw data generated in this study are available at NCBI/SRA under the BioProject number PRJNA1294573. Source codes to analyze the data and generate the figures are available at github.com/ecomicroaqua/San_Quintin.
