# Supplementary material for: Taxonomic and functional stability of sedimentary microbial communities in a pristine upwelling-influenced coastal lagoon
Source: ISME Commun. 2025 Dec 18;5(1):ycaf241. doi: 10.1093/ismeco/ycaf241 (PMC12766710; doi:10.1093/ismeco/ycaf241)
Supplement: Supplemental_figures_ycaf241 [file supplemental_figures_ycaf241.pdf]

SUPPLEMENTAL FIGURES

Taxonomic and functional stability of sedimentary microbial communities in a pristine upwelling-influenced coastal lagoon

Jorge Rojas-Vargas[1,2,3], Guillermo Samperio-Ramos[4], Víctor F. Camacho-Ibar[4], Silvia Pajares[1]*

[1] Unidad Académica de Ecología y Biodiversidad Acuática, Institute of Marine Sciences and Limnology, National Autonomous University of Mexico (UNAM), Mexico City, Mexico.

[2] Department of Biology, University of Western Ontario (UWO), London, Ontario, Canada.

[3] Department of Microbiology & Immunology, Schulich School of Medicine & Dentistry, University of Western Ontario (UWO), London, Ontario, Canada.

[4] Institute of Oceanological Research, Autonomous University of Baja California (UABC), Ensenada, Mexico.

* Corresponding author e-mail: spajares@cmarl.unam.mx

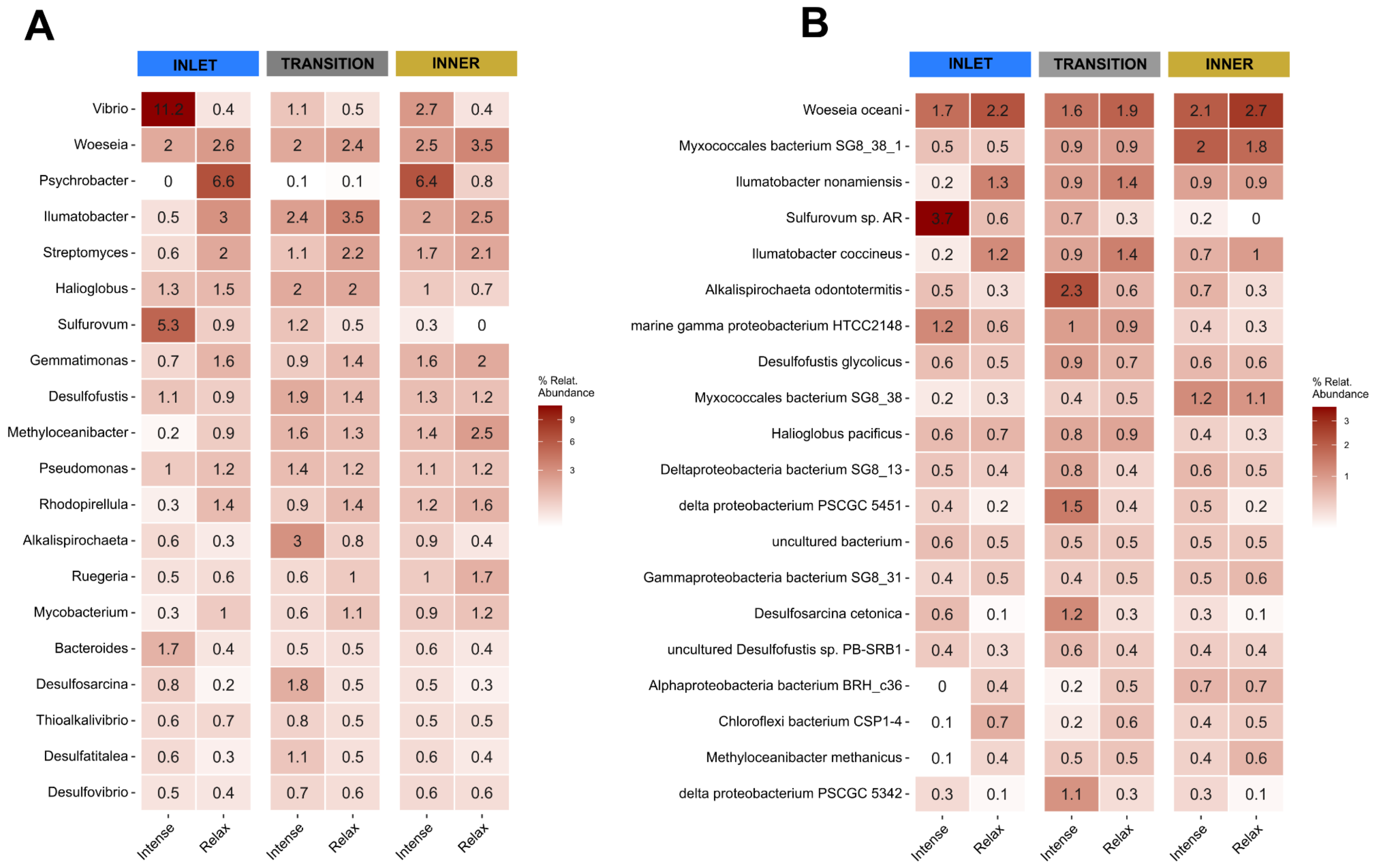

Fig. S1. Mean relative abundances (%) of the top 20 most abundant taxa across upwelling season and sectors, shown at the genus (**A**) and species level (**B**).

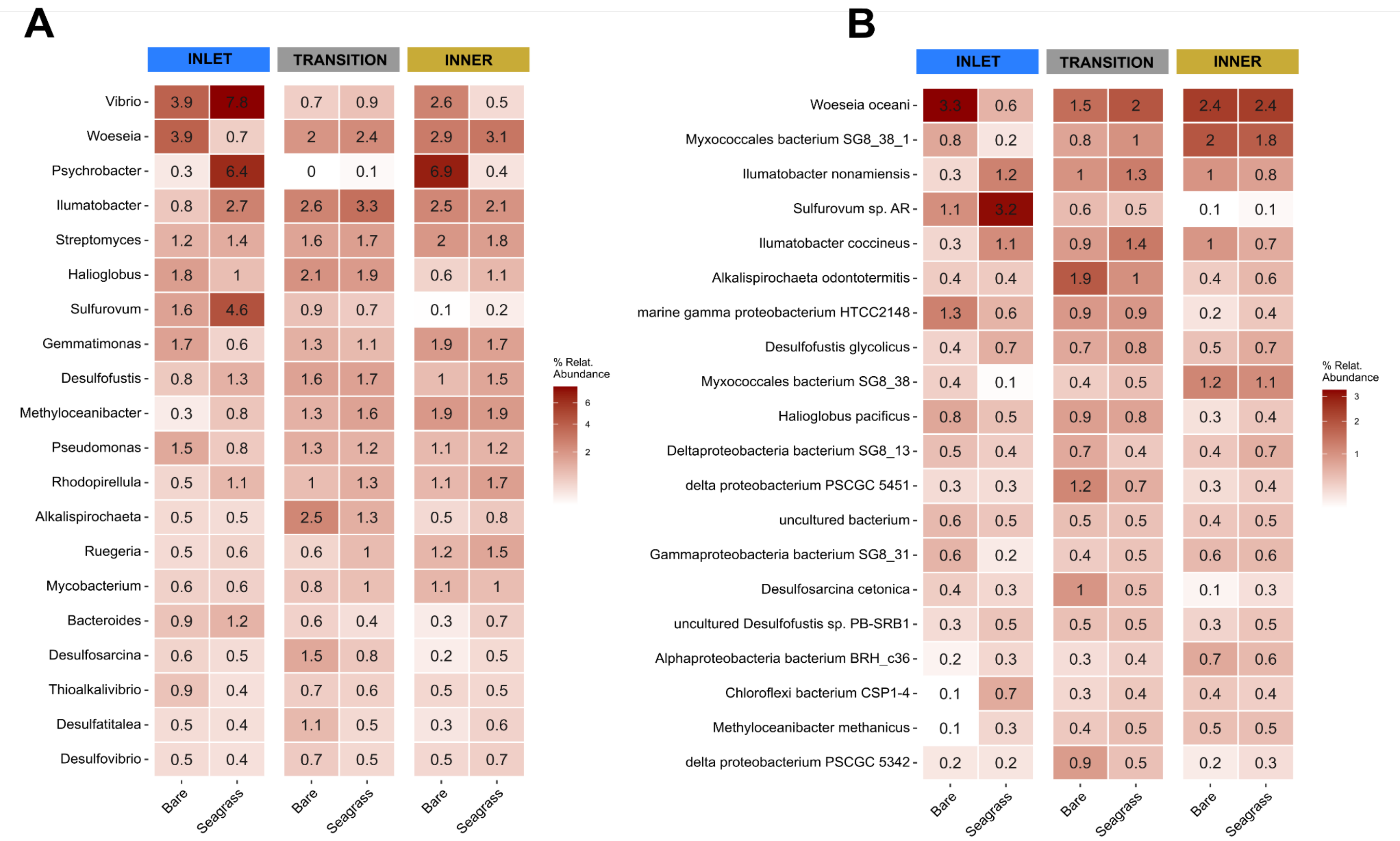

Fig. S2. Mean relative abundances (%) of the top 20 most abundant taxa across habitats and sectors, shown at the genus (**A**) and species level (**B**).

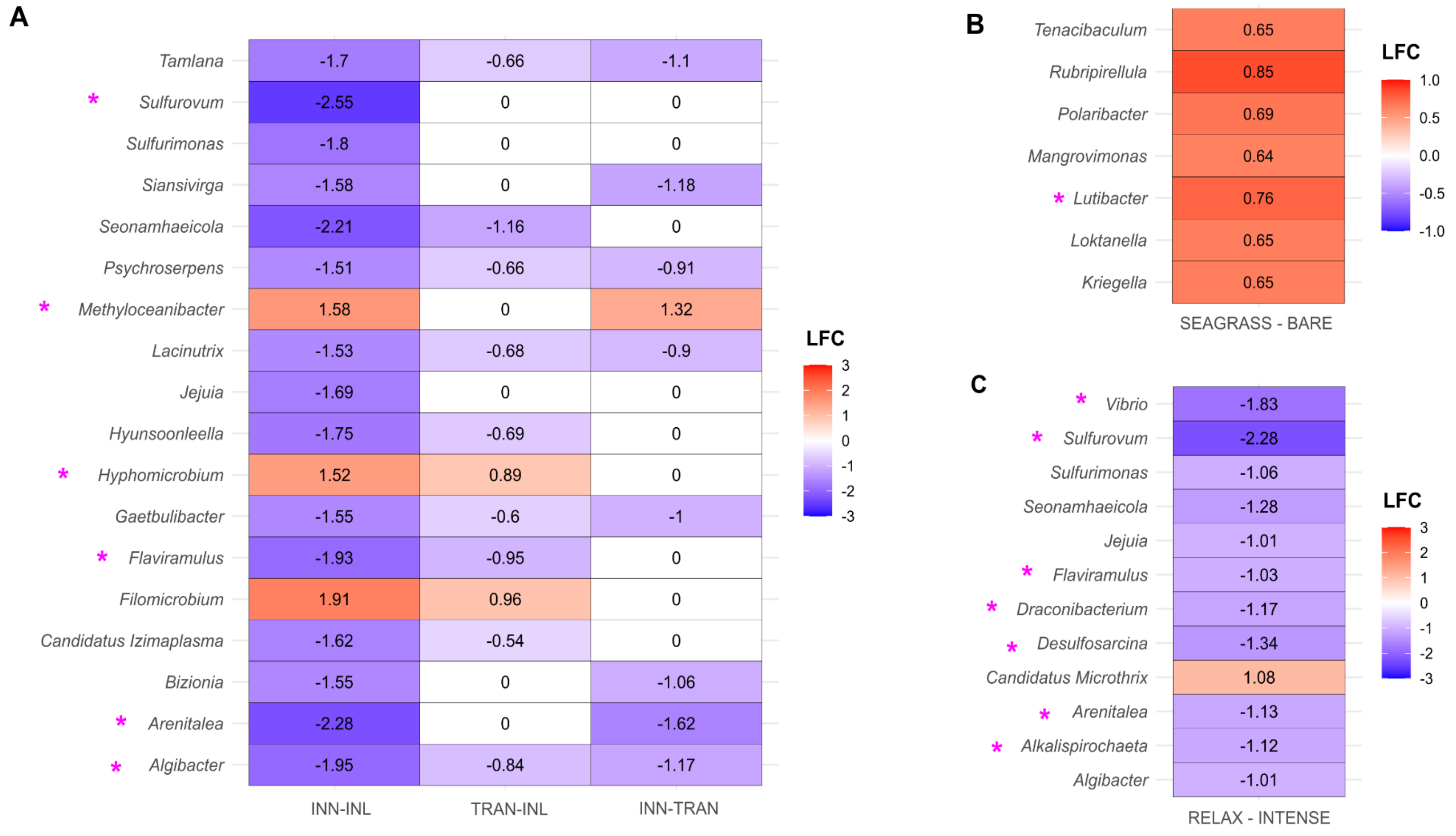

Fig. S3. Differential abundance of bacterial genera across sectors (**A**), habitats (**B**), and upwelling seasons (**C**). The y-axis shows genera, and the x-axis indicates pairwise comparisons between levels within each factor. Data are presented as effect sizes (log fold change, LFC) with 95% confidence intervals, calculated from beta coefficients and standard errors estimated using the ANCOM-BC model (two-sided; FDR-adjusted). Only genera with FDR $q < 0.05$ are plotted. Colors indicate LFC direction: red denotes significantly higher abundance in the first level of the comparison, blue in the second. Only genera with relative abundance (RA) ≥0.1% in at least one sample are shown. Pink asterisks mark genera with RA ≥0.5%. Abbreviations of sectors: INN = inner, INL = inlet, TRAN = transition.

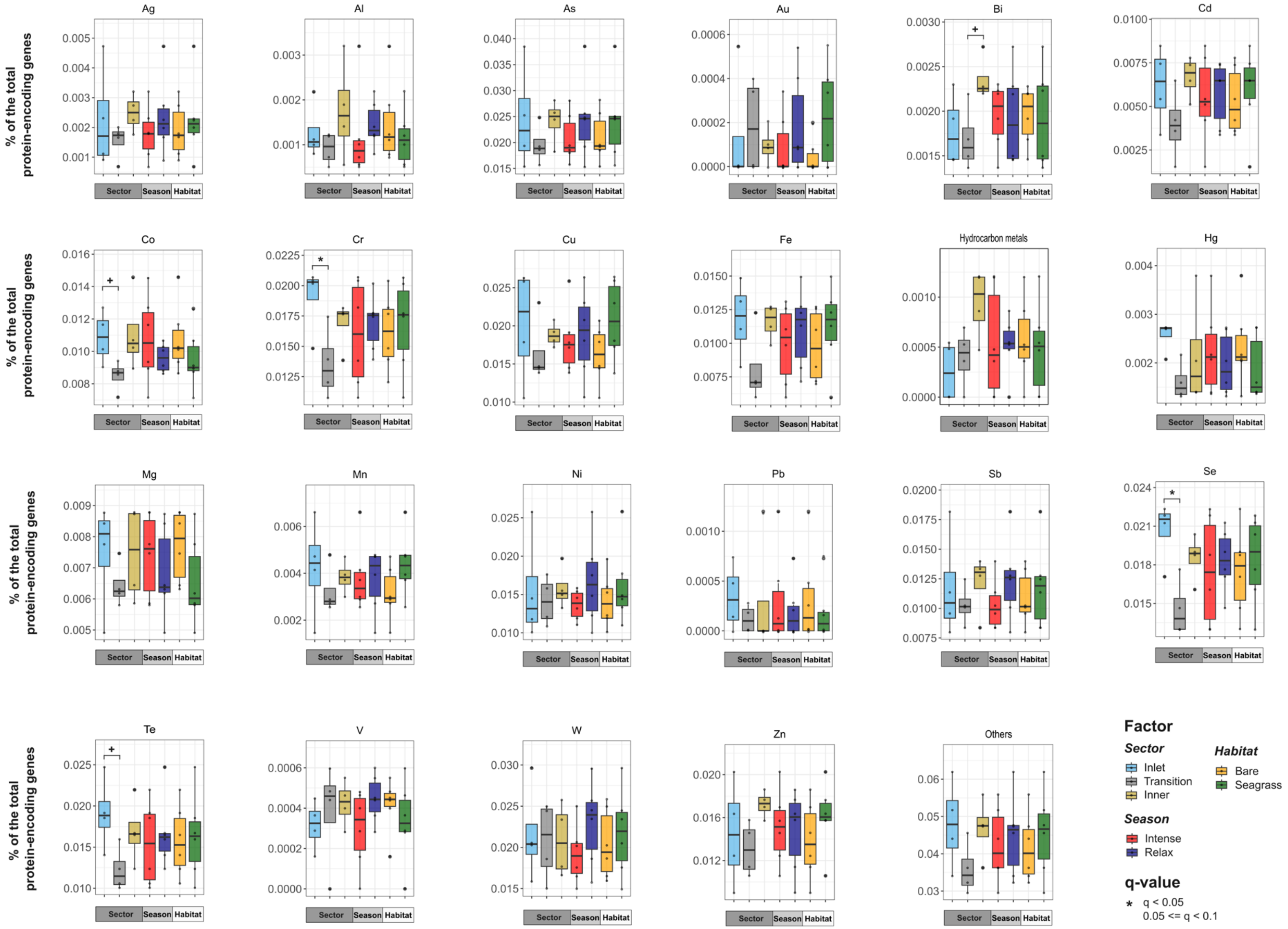

Fig. S4. Distribution of metal-associated genes across lagoon sectors, upwelling seasons, and habitats. Asterisks (*) denote significant differences ($q < 0.05$), and crosses ($^{+}$) indicate marginal significance ($0.05 < q < 0.10$) based on FDR-adjusted Dunn tests for sectors and Wilcoxon tests for seasons and habitats.

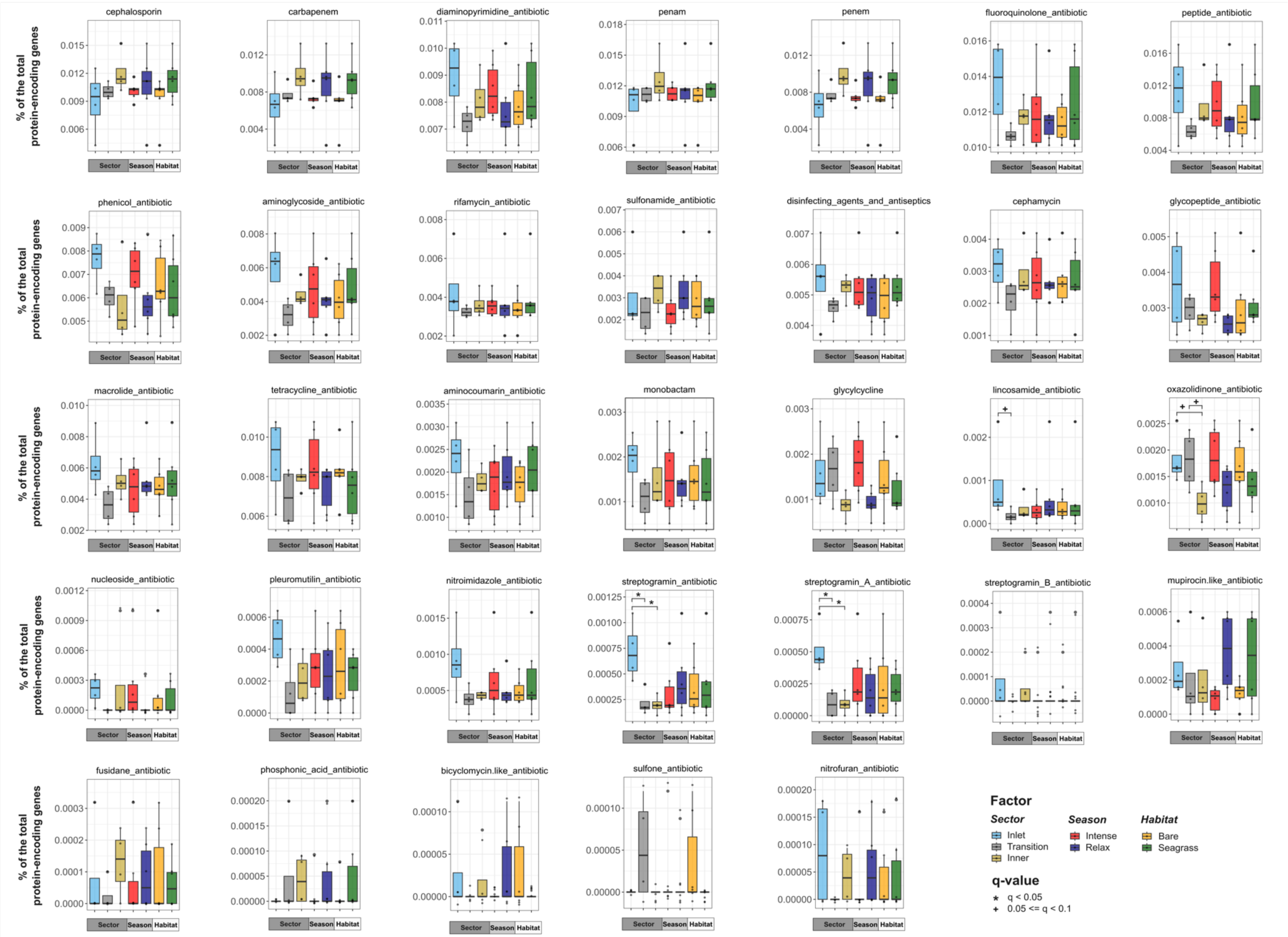

Fig. S5. Distribution of genes related to antibiotic resistance drug classes across lagoon sectors, upwelling seasons, and habitats. Asterisks (*) denote significant differences ($q < 0.05$), and crosses ($^+$) indicate marginal significance ($0.05 < q < 0.10$) based on FDR-adjusted Dunn tests for sectors and Wilcoxon tests for seasons and habitats.

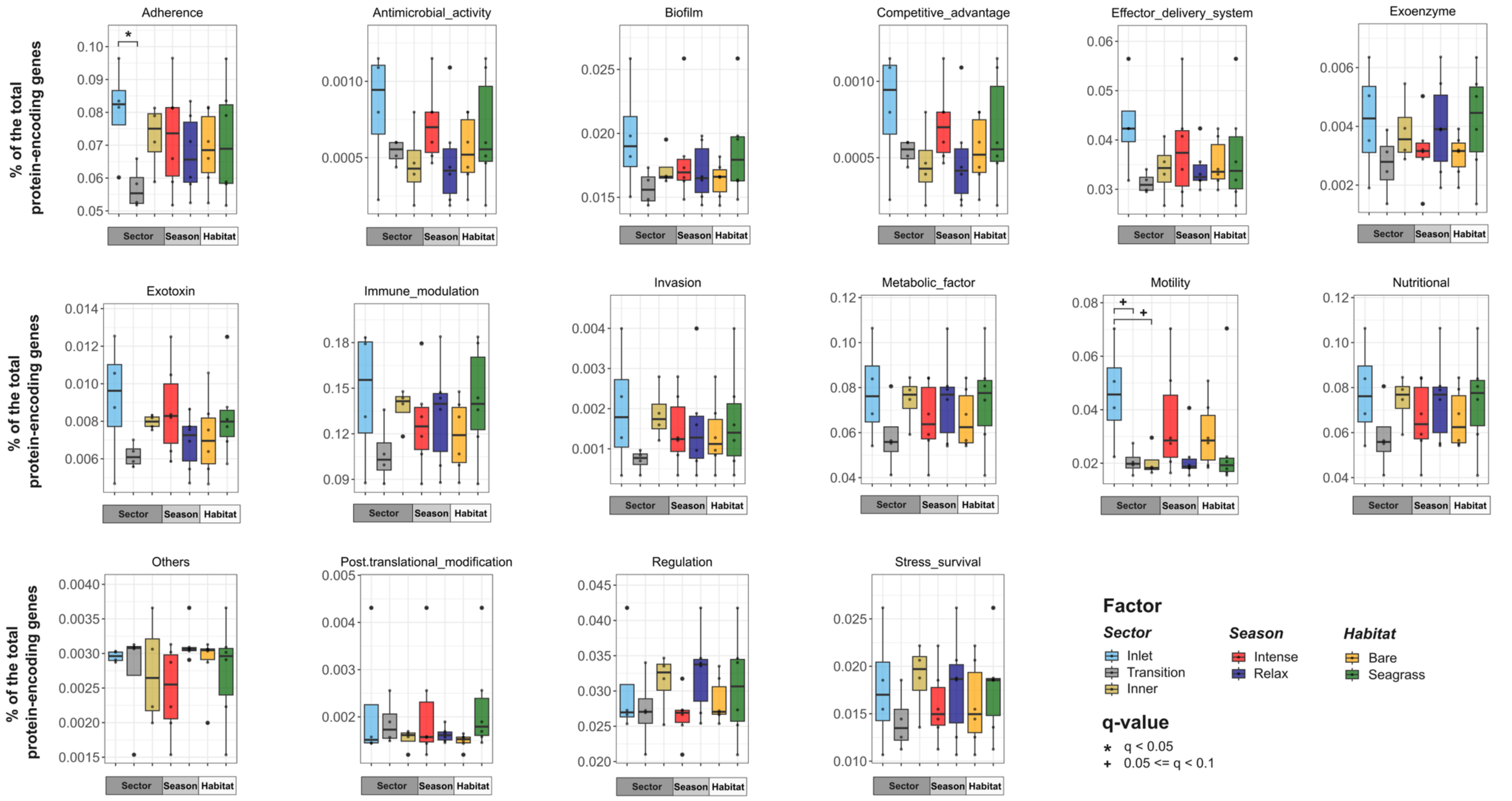

Fig. S6. Distribution of genes related to virulence factors across lagoon sectors, upwelling seasons, and habitats. Asterisks (*) denote significant differences ($q < 0.05$), and crosses ($^{+}$) indicate marginal significance ($0.05 < q < 0.10$) based on FDR-adjusted Dunn tests for sectors and Wilcoxon tests for seasons and habitats.

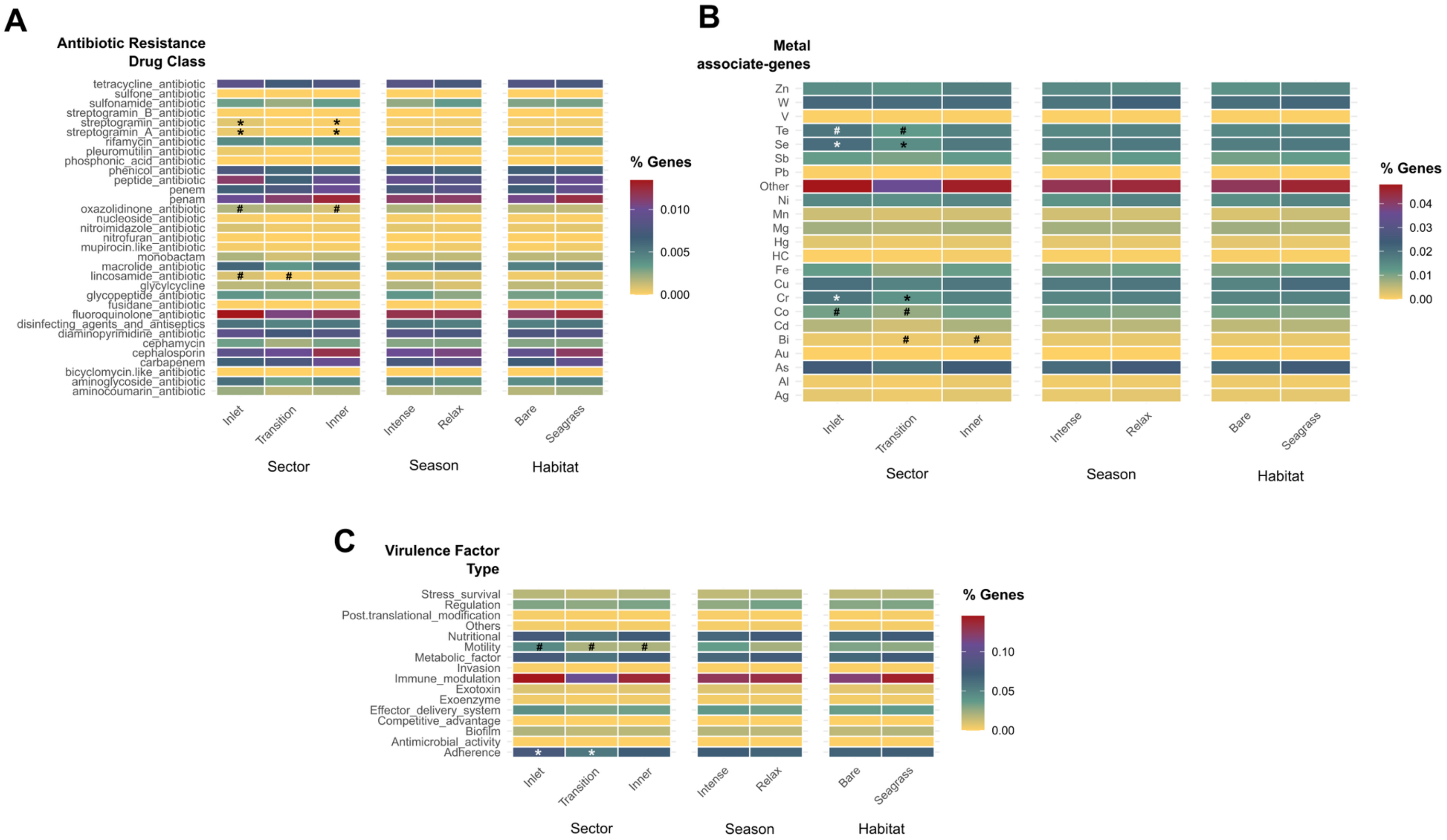

Fig. S7. Heatmaps comparing the average gene content (%) related to antibiotic resistance drug classes (**A**), metal-associated genes (**B**), and virulence factor types (**C**). Each row represents a specific resistance class, metal type, or virulence category, while columns correspond to environmental factors: sector (inlet, transition, inner), season (intense, relaxed), and habitat (bare, seagrass). Color intensity reflects the mean percentage of gene content. Asterisks (*) indicate significant differences ($q < 0.05$); crosshatches (#) indicate marginal significance ($0.05 < q < 0.10$) based on FDR-adjusted Dunn tests for sectors and Wilcoxon tests for seasons and habitats.

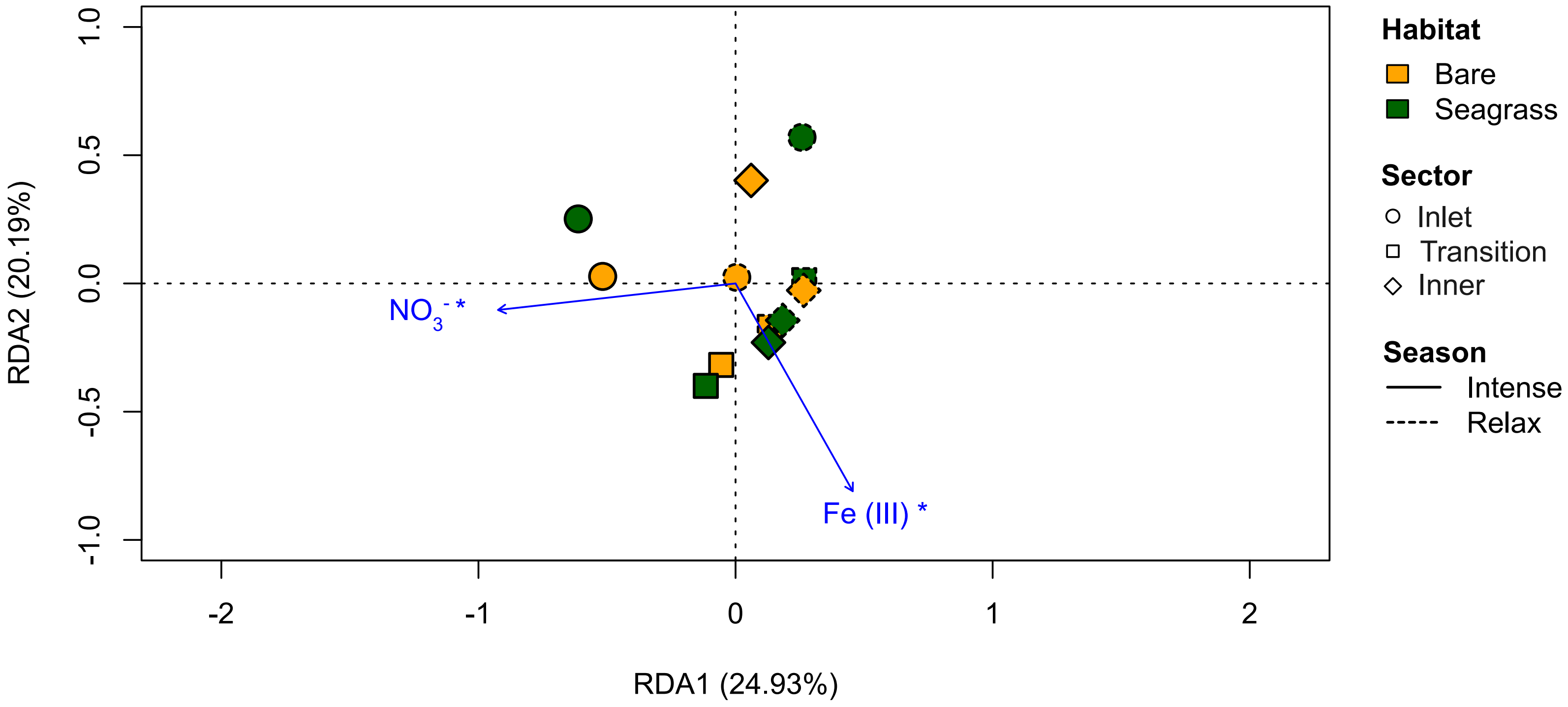

Fig. S8. Redundancy Analysis (RDA) plot depicting the relationship between the predicted functional-gene counts and the physicochemical factors. Length and angle of arrows: extent of correlation between significant environmental factors ($p < 0.05$, 999 permutations) and RDA axes.
