# Supplementary material for: Taxonomic and functional stability of sedimentary microbial communities in a pristine upwelling-influenced coastal lagoon
Source: ISME Commun. 2025 Dec 18;5(1):ycaf241. doi: 10.1093/ismeco/ycaf241 (PMC12766710; doi:10.1093/ismeco/ycaf241)
Supplement: Supplemental_tables_ycaf241 [file supplemental_tables_ycaf241.pdf]

SUPPLEMENTAL TABLES

Taxonomic and functional stability of sedimentary microbial communities in a pristine upwelling-influenced coastal lagoon

Jorge Rojas-Vargas[1,2,3], Guillermo Samperio-Ramos[4], Víctor F. Camacho-Ibar[4], Silvia Pajares[1]*

[1] Unidad Académica de Ecología y Biodiversidad Acuática, Institute of Marine Sciences and Limnology, National Autonomous University of Mexico (UNAM), Mexico City, Mexico.

[2] Department of Biology, University of Western Ontario (UWO), London, Ontario, Canada.

[3] Department of Microbiology & Immunology, Schulich School of Medicine & Dentistry, University of Western Ontario (UWO), London, Ontario, Canada.

[4] Institute of Oceanological Research, Autonomous University of Baja California (UABC), Ensenada, Mexico.

* Corresponding author e-mail: spajares@cmarl.unam.mx

Table S1. Quality control and metagenome assembly features.

FACTORS			QUALITY (Trimmomatic)					ASSEMBLY (MEGAHIT)					
Sector	Habitat	Upwelling Season	Input read pairs	Both surviving		Dropped		Contigs	Size [bp]	min [bp]	max [bp]	avg [bp]	N50 [bp]
Inlet	Bare	Relax	64,163,470	38,194,317	59.53%	454,249	0.71%	724,297	360,060,933	200	135,410	497	470
Inlet	Seagrass	Relax	52,069,927	36,030,518	69.20%	330,271	0.63%	405,534	270,879,910	200	219,320	668	701
Inlet	Bare	Intense	54,431,906	36,914,599	67.82%	384,384	0.71%	512,076	268,552,135	200	20,855	524	493
Inlet	Seagrass	Intense	42,925,070	32,417,068	75.52%	204,200	0.48%	555,972	319,088,224	200	55,133	574	561
Transition	Bare	Relax	78,871,953	47,306,593	59.98%	452,303	0.57%	929,148	481,660,421	200	100,901	518	491
Transition	Seagrass	Relax	44,479,298	35,385,950	79.56%	227,691	0.51%	756,483	461,143,336	200	108,144	610	606
Transition	Bare	Intense	55,640,238	41,156,651	73.97%	318,987	0.57%	679,311	352,581,300	200	73,154	519	492
Transition	Seagrass	Intense	45,353,543	28,116,367	61.99%	287,530	0.63%	492,005	236,320,183	200	55,085	480	450
Inner	Bare	Relax	55,939,922	46,332,428	82.83%	247,496	0.44%	970,992	591,720,503	200	168,907	609	603
Inner	Seagrass	Relax	52,009,291	38,407,962	73.85%	283,030	0.54%	820,740	482,408,350	200	97,342	588	572
Inner	Bare	Intense	42,925,070	32,417,068	75.52%	204,200	0.48%	402,223	216,015,949	200	202,102	537	499
Inner	Seagrass	Intense	52,390,412	36,706,559	70.06%	287,550	0.55%	853,209	470,583,584	200	119,143	552	524
**Average**			53,433,342	37,448,840	70.82%	306,824	0.57%	675,166	375,917,902	200	112,958	556	539
**Standard deviation**			10,205,565	5,528,052	7.50%	86,217	0.09%	198,976	119,453,156	0	60,381	55	72

Table S2. Three-factor aligned-rank-transform ANOVA (ART) for sediment physicochemical variables—F statistics and p-values for sector, upwelling season, habitat, and their interactions.

Response	Term	Df	Df.res	F value	p-value
Sand	Sector	2	24	87	0.00000
	Season	1	24	13.05	0.00139
	Habitat	1	24	36.72	0.00000
	Sector:Season	2	24	11.76	0.00028
	Sector:Habitat	2	24	10.12	0.00065
	Season:Habitat	1	24	0.96	0.33700
	Sector:Season:Habitat	2	24	37.14	0.00000
Silt	Sector	2	24	82.32	0.00000
	Season	1	24	13.92	0.00104
	Habitat	1	24	37.29	0.00000
	Sector:Season	2	24	11.67	0.00029
	Sector:Habitat	2	24	9.95	0.00071
	Season:Habitat	1	24	0.96	0.33700
	Sector:Season:Habitat	2	24	31.69	0.00000
pH	Sector	2	24	54.75	0.00000
	Season	1	24	39.72	0.00000
	Habitat	1	24	0.16	0.69500
	Sector:Season	2	24	3.95	0.03290
	Sector:Habitat	2	24	0.67	0.52000
	Season:Habitat	1	24	0.04	0.83500
	Sector:Season:Habitat	2	24	2.25	0.12700
TOC	Sector	2	24	46.45	0.00000
	Season	1	24	47.52	0.00000
	Habitat	1	24	56.72	0.00000
	Sector:Season	2	24	33.76	0.00000
	Sector:Habitat	2	24	0.91	0.41500
	Season:Habitat	1	24	0.83	0.37200
	Sector:Season:Habitat	2	24	0.92	0.41300
TN	Sector	2	24	36.77	0.00000
	Season	1	24	61.45	0.00000
	Habitat	1	24	32.43	0.00001
	Sector:Season	2	24	30.22	0.00000
	Sector:Habitat	2	24	0.15	0.86200
	Season:Habitat	1	24	0.18	0.67100
	Sector:Season:Habitat	2	24	0.63	0.54100
NH_4^+	Sector	2	24	18.37	0.00001
	Season	1	24	37.8	0.00000
	Habitat	1	24	73.1	0.00000
	Sector:Season	2	24	19.23	0.00001
	Sector:Habitat	2	24	11.94	0.00025
	Season:Habitat	1	24	1.81	0.19100
	Sector:Season:Habitat	2	24	9.5	0.00091
NO_3^-	Sector	2	24	7.74	0.00255
	Season	1	24	40	0.00000
	Habitat	1	24	0.03	0.87400
	Sector:Season	2	24	10.11	0.00065
	Sector:Habitat	2	24	3.11	0.06280
	Season:Habitat	1	24	2.19	0.15200

	Sector:Season:Habitat	2	24	0.04	0.96300
NO_2^-	Sector	2	24	4.15	0.02840
	Season	1	24	4.52	0.04390
	Habitat	1	24	0.87	0.36100
	Sector:Season	2	24	2.77	0.08270
	Sector:Habitat	2	24	2.18	0.13500
	Season:Habitat	1	24	1.72	0.20200
	Sector:Season:Habitat	2	24	0.62	0.54700
Fe(III)	Sector	2	24	9.36	0.00099
	Season	1	24	0	0.97900
	Habitat	1	24	0.55	0.46400
	Sector:Season	2	24	0.24	0.78500
	Sector:Habitat	2	24	2.22	0.13000
	Season:Habitat	1	24	1.48	0.23500
	Sector:Season:Habitat	2	24	0.03	0.97100
Fe(II)	Sector	2	24	9.96	0.00071
	Season	1	24	0.38	0.54400
	Habitat	1	24	2.35	0.13900
	Sector:Season	2	24	1.69	0.20700
	Sector:Habitat	2	24	0.14	0.87000
	Season:Habitat	1	24	0.21	0.65400
	Sector:Season:Habitat	2	24	0.74	0.49000

Table S3. Relative abundance (%) of prokaryotic classes in San Quintín sediments. Abbreviation: A_: Archaea, B_: Bacteria.

**Sector**	Inlet				Transition				Inner			
**Upwelling Season**	Relax		Intense		Relax		Intense		Relax		Intense	
**Habitat**	Bare	Seagrass	Bare	Seagrass	Bare	Seagrass	Bare	Seagrass	Bare	Seagrass	Bare	Seagrass
A_Archaeoglobi	0.0114	0.0139	0.0171	0.0121	0.0219	0.0181	0.0109	0.0086	0.0117	0.0134	0.0139	0.0229
A_Candidatus Thalassoarchaea	0.0036	0.0016	0.0030	0.0048	0.0036	0.0010	0.0008	0.0023	0.0016	0.0019	0.0023	0.0028
A_Hadesarchaea	0.0059	0.0051	0.0091	0.0058	0.0081	0.0066	0.0053	0.0023	0.0067	0.0072	0.0060	0.0099
A_Halobacteria	0.2156	0.2186	0.2284	0.2461	0.2855	0.2224	0.0978	0.0785	0.1467	0.1354	0.1954	0.2379
A_Methanobacteria	0.0273	0.0293	0.0374	0.0325	0.0418	0.0341	0.0277	0.0229	0.0303	0.0296	0.0240	0.0472
A_Methanococci	0.0082	0.0083	0.0112	0.0091	0.0096	0.0088	0.0070	0.0093	0.0076	0.0037	0.0060	0.0105
A_Methanomicrobia	0.1496	0.1992	0.2419	0.1770	0.2453	0.2189	0.1805	0.1382	0.2044	0.1609	0.1650	0.2557
A_Methanonatronarchaeia	0.0000	0.0012	0.0016	0.0006	0.0012	0.0012	0.0008	0.0008	0.0013	0.0009	0.0004	0.0012
A_Methanopyri	0.0014	0.0004	0.0004	0.0011	0.0017	0.0008	0.0003	0.0003	0.0007	0.0006	0.0008	0.0010
A_Nanohaloarchaea	0.0014	0.0032	0.0012	0.0013	0.0015	0.0020	0.0006	0.0003	0.0004	0.0003	0.0004	0.0008
A_Nitrososphaeria	0.0091	0.0055	0.0048	0.0039	0.0077	0.0080	0.0025	0.0030	0.0040	0.0059	0.0068	0.0069
A_Theionarchaea	0.0098	0.0135	0.0258	0.0113	0.0207	0.0172	0.0154	0.0131	0.0193	0.0137	0.0120	0.0261
A_Thermococci	0.0196	0.0273	0.0305	0.0197	0.0310	0.0240	0.0219	0.0156	0.0215	0.0221	0.0195	0.0316
A_Thermoplasmata	0.0303	0.0246	0.0390	0.0286	0.0454	0.0357	0.0322	0.0322	0.0321	0.0286	0.0353	0.0509
A_Thermoprotei	0.0426	0.0345	0.0419	0.0383	0.0432	0.0400	0.0233	0.0211	0.0294	0.0277	0.0353	0.0438
B_Acidimicrobiia	0.9031	4.6797	2.3411	4.1707	2.5459	2.2495	0.6502	0.2381	1.9292	1.8904	2.1054	1.5177
B_Acidithiobacillia	0.5323	0.1323	0.3115	0.2532	0.2730	0.3757	0.3453	0.2225	0.3377	0.3647	0.3686	0.2533
B_Acidobacteriia	0.3340	0.2689	0.3194	0.3125	0.3994	0.3293	0.1348	0.1326	0.2037	0.2035	0.2826	0.3651
B_Actinobacteria	7.7457	12.3111	8.9127	13.1104	11.6046	8.8253	3.3793	2.0918	5.4500	4.8178	7.7415	7.4241
B_Alphaproteobacteria	16.9035	15.7343	12.8509	20.7816	21.4519	18.5885	10.1261	9.9153	11.0665	13.8629	15.7866	14.9842
B_Anaerolineae	0.7012	1.3523	1.6927	0.9627	1.8395	1.6307	1.0283	0.8886	1.0336	0.8806	0.8812	2.2935
B_Aquificae	0.0514	0.0420	0.0582	0.0485	0.0685	0.0570	0.0362	0.0388	0.0453	0.0461	0.0522	0.0729
B_Ardenticatenia	0.1666	0.2823	0.2798	0.2922	0.4556	0.5195	0.1037	0.0793	0.2585	0.1596	0.1710	0.5712
B_Bacilli	1.3955	3.6747	1.6852	1.5836	1.7288	1.5780	1.0838	1.2880	1.2871	1.7814	3.5277	1.7424
B_Bacteroidia	0.6739	1.4584	1.4578	0.9731	0.7223	1.0096	3.8207	4.5742	1.2135	1.3309	0.6613	1.7029
B_Balneolia	0.1029	0.0554	0.0823	0.0913	0.0981	0.0944	0.0771	0.0607	0.0671	0.0647	0.0695	0.0956
B_Betaproteobacteria	7.0893	2.8946	4.8264	4.2255	4.5993	4.7148	4.2992	3.1201	4.7466	4.6352	4.4870	4.4093
B_Blastocatellia	0.0610	0.0451	0.0577	0.0567	0.0851	0.0628	0.0266	0.0247	0.0350	0.0333	0.0549	0.0648
B_Caldilineae	0.0815	0.1600	0.1929	0.1597	0.1631	0.2046	0.0409	0.0420	0.1162	0.0958	0.0894	0.2097

B_Caldisericia	0.0039	0.0051	0.0057	0.0037	0.0081	0.0035	0.0059	0.0025	0.0045	0.0028	0.0034	0.0045
B_Calditrichae	0.0569	0.0554	0.0841	0.0543	0.0778	0.0767	0.0824	0.0873	0.0545	0.0563	0.0556	0.1173
B_Candidatus Lambdaproteobacteria	0.0148	0.0123	0.0194	0.0154	0.0170	0.0170	0.0126	0.0121	0.0114	0.0134	0.0116	0.0148
B_Candidatus Muproteobacteria	0.4733	0.1129	0.2414	0.1634	0.1771	0.2064	0.2455	0.1850	0.2686	0.3059	0.2296	0.1862
B_Candidatus Peribacteria	0.0130	0.0103	0.0109	0.0095	0.0164	0.0121	0.0050	0.0048	0.0079	0.0087	0.0079	0.0154
B_Chitinispirillia	0.0127	0.0135	0.0151	0.0087	0.0115	0.0103	0.0149	0.0096	0.0087	0.0121	0.0041	0.0142
B_Chitinivibrionia	0.0032	0.0040	0.0059	0.0061	0.0058	0.0060	0.0056	0.0038	0.0043	0.0047	0.0068	0.0077
B_Chitinophagia	0.1962	0.2186	0.2209	0.2474	0.1927	0.2005	0.3176	0.3209	0.2080	0.2247	0.1405	0.2597
B_Chlamydiia	0.0487	0.0539	0.0579	0.0537	0.0634	0.0529	0.0314	0.0430	0.0449	0.0451	0.0417	0.0604
B_Chlorobia	0.1061	0.0883	0.1165	0.0991	0.1012	0.1044	0.0956	0.0903	0.0983	0.1011	0.0864	0.1171
B_Chloroflexia	0.1487	0.1639	0.1774	0.1508	0.2090	0.1740	0.0762	0.0657	0.1059	0.1005	0.1315	0.2026
B_Chrysiogenetes	0.0173	0.0135	0.0192	0.0128	0.0183	0.0187	0.0160	0.0126	0.0204	0.0177	0.0162	0.0237
B_Chthonomonadetes	0.0105	0.0147	0.0119	0.0139	0.0156	0.0121	0.0045	0.0063	0.0108	0.0075	0.0083	0.0134
B_Clostridia	1.2719	1.6964	1.9367	1.4799	1.8388	1.6420	1.6211	2.1734	1.5186	1.3785	1.3869	2.0459
B_Coriobacteriia	0.0779	0.2891	0.1934	0.1688	0.2369	0.1797	0.0426	0.0440	0.0891	0.0722	0.1090	0.1250
B_Cytophagia	1.0661	1.1994	1.3067	1.7896	1.1242	1.2284	1.8557	1.9977	1.3470	1.5079	0.8338	1.6219
B_Deferribacteres	0.0453	0.0337	0.0533	0.0305	0.0486	0.0476	0.0482	0.0315	0.0455	0.0405	0.0421	0.0626
B_Dehalococcoidia	0.0562	0.0816	0.1154	0.0719	0.1489	0.1075	0.0603	0.0526	0.0844	0.0675	0.0879	0.1238
B_Deinococci	0.2866	0.2831	0.2805	0.3088	0.3383	0.2757	0.1247	0.1062	0.1784	0.1668	0.2409	0.2814
B_Deltaproteobacteria	9.8931	10.6048	20.1193	9.7041	15.3493	17.0165	19.1920	12.8233	26.9913	22.2389	15.2320	22.5360
B_Dictyoglomia	0.0046	0.0051	0.0055	0.0043	0.0077	0.0064	0.0031	0.0030	0.0043	0.0037	0.0045	0.0061
B_Elusimicrobia	0.0025	0.0028	0.0023	0.0028	0.0029	0.0035	0.0020	0.0020	0.0038	0.0037	0.0045	0.0041
B_Endomicrobia	0.0030	0.0008	0.0036	0.0024	0.0038	0.0033	0.0034	0.0025	0.0025	0.0019	0.0019	0.0024
B_Epsilonproteobacteria	0.1915	2.0116	0.9954	0.1736	0.1634	0.1713	3.2742	8.0301	0.9849	1.4423	0.2818	0.5481
B_Erysipelotrichia	0.0225	0.0305	0.0297	0.0238	0.0296	0.0240	0.0230	0.0287	0.0193	0.0233	0.0180	0.0316
B_Fibrobacteria	0.0157	0.0154	0.0175	0.0145	0.0166	0.0133	0.0149	0.0159	0.0094	0.0131	0.0105	0.0194
B_Fimbriimonadia	0.0137	0.0154	0.0144	0.0193	0.0146	0.0164	0.0070	0.0063	0.0090	0.0081	0.0116	0.0144
B_Flavobacteriia	2.2467	5.4095	2.1702	4.1794	2.3263	2.8396	9.0204	13.6523	3.1946	4.0561	1.5861	2.7267
B_Fusobacteriia	0.0362	0.0341	0.0422	0.0329	0.0360	0.0363	0.0462	0.0501	0.0406	0.0386	0.0353	0.0448
B_Gammaproteobacteria	37.0940	23.2263	26.3095	21.8151	19.0675	24.5418	32.6780	31.8491	26.6236	29.4372	35.3933	20.5859
B_Gemmatimonadetes	1.9223	0.6553	1.2823	1.0204	1.6850	1.4071	0.8195	0.3971	0.7101	0.7347	1.3351	1.2175
B_Gloeobacteria	0.0282	0.0253	0.0253	0.0212	0.0308	0.0250	0.0107	0.0103	0.0177	0.0118	0.0162	0.0243
B_Holophagae	0.0430	0.0392	0.0545	0.0409	0.0507	0.0482	0.0255	0.0244	0.0307	0.0342	0.0395	0.0594

B_Hydrogenophilalia	0.0683	0.0277	0.0513	0.0420	0.0373	0.0445	0.0519	0.0415	0.0550	0.0601	0.0556	0.0446
B_Ignavibacteria	0.2465	0.2035	0.3122	0.2361	0.3159	0.3016	0.4863	0.4225	0.2401	0.2555	0.2454	0.4093
B_Kiritimatiellae	0.0319	0.0420	0.0406	0.0392	0.0355	0.0341	0.0314	0.0425	0.0332	0.0349	0.0274	0.0464
B_Ktedonobacteria	0.0681	0.0875	0.0973	0.0809	0.0985	0.0821	0.0364	0.0393	0.0628	0.0616	0.0654	0.0944
B_Lentisphaeria	0.0601	0.0970	0.0600	0.1041	0.0471	0.0507	0.0294	0.0325	0.0770	0.0523	0.0267	0.0602
B_Limnochordia	0.0214	0.0249	0.0203	0.0199	0.0315	0.0217	0.0056	0.0063	0.0101	0.0100	0.0188	0.0176
B_Methylacidiphilae	0.0096	0.0067	0.0064	0.0093	0.0094	0.0070	0.0034	0.0038	0.0040	0.0081	0.0034	0.0099
B_Mollicutes	0.0200	0.0210	0.0210	0.0173	0.0195	0.0193	0.0185	0.0249	0.0197	0.0165	0.0158	0.0199
B_Negativicutes	0.1300	0.1667	0.1901	0.1541	0.1906	0.1738	0.1256	0.1070	0.1517	0.1375	0.1330	0.1870
B_Nitriliruptoria	0.0829	0.0950	0.0750	0.1141	0.1119	0.0913	0.0249	0.0151	0.0590	0.0510	0.0658	0.0650
B_Nitrospinia	0.0863	0.0127	0.0285	0.0195	0.0265	0.0297	0.0151	0.0101	0.0222	0.0202	0.0244	0.0334
B_Nitrospira	0.5184	0.3041	0.6929	0.3391	1.1201	0.7117	0.4058	0.2987	0.7915	0.6413	0.4791	0.8291
B_Oligoflexia	0.1773	0.1129	0.1689	0.1465	0.1615	0.1508	0.1135	0.0974	0.1382	0.1316	0.1428	0.1779
B_Opitutae	0.2117	0.3132	0.2485	0.3259	0.2227	0.2044	0.1586	0.1397	0.1898	0.2144	0.1879	0.3047
B_Phycisphaerae	0.2736	0.4023	0.4539	0.3666	0.3619	0.3328	0.2251	0.1918	0.3560	0.2745	0.2379	0.4779
B_Planctomycetia	2.7655	4.7542	2.4007	5.1628	3.9782	3.6824	0.7015	1.0832	2.6178	1.6318	1.7522	4.2098
B_Rubrobacteria	0.0512	0.0673	0.0586	0.0751	0.0903	0.0634	0.0238	0.0166	0.0343	0.0302	0.0556	0.0604
B_Saprospiria	0.1498	0.1164	0.1249	0.1580	0.1089	0.1206	0.1889	0.2293	0.1122	0.1394	0.0812	0.1404
B_Solibacteres	0.1275	0.0958	0.1291	0.1167	0.1535	0.1235	0.0390	0.0445	0.0792	0.0629	0.0939	0.1434
B_Spartobacteria	0.0808	0.1200	0.0506	0.1519	0.0713	0.0589	0.0244	0.0249	0.0485	0.0423	0.0413	0.0705
B_Sphingobacteriia	0.2636	0.3433	0.3222	0.3733	0.2720	0.2957	0.5199	0.5335	0.3020	0.3043	0.2040	0.3862
B_Spirochaetia	0.5284	0.4867	1.5041	0.3740	0.5570	0.5852	0.7363	0.7381	2.6543	2.0347	0.6786	1.1676
B_Synergistia	0.0669	0.0788	0.0976	0.0799	0.0985	0.0919	0.0650	0.0534	0.0749	0.0666	0.0748	0.1080
B_Thermodesulfobacteria	0.0414	0.0337	0.0517	0.0305	0.0582	0.0462	0.0297	0.0322	0.0487	0.0457	0.0421	0.0610
B_Thermoflexia	0.0000	0.0012	0.0007	0.0006	0.0009	0.0004	0.0003	0.0003	0.0002	0.0006	0.0000	0.0004
B_Thermoleophilia	0.1625	0.4007	0.2994	0.4906	0.7085	0.5160	0.0597	0.0433	0.1229	0.1214	0.3934	0.3080
B_Thermomicrobia	0.0603	0.0867	0.0787	0.0770	0.1038	0.0792	0.0238	0.0226	0.0471	0.0373	0.0628	0.0863
B_Thermotogae	0.0628	0.0673	0.0916	0.0714	0.0942	0.0841	0.0737	0.0720	0.0666	0.0632	0.0725	0.0896
B_Tissierellia	0.0553	0.0558	0.0698	0.0580	0.0668	0.0681	0.0720	0.0939	0.0556	0.0461	0.0575	0.0701
B_Verrucomicrobiae	0.7009	1.2070	0.4074	1.3232	0.5132	0.4147	0.2124	0.2051	0.3175	0.4434	0.3382	0.6404
B_Zetaproteobacteria	0.1730	0.0649	0.1400	0.0853	0.0976	0.1075	0.1329	0.1248	0.1472	0.1606	0.1060	0.1066

Table S4. Stepwise permutation test (ordistep function, 999 permutations) used to select environmental predictors for the redundancy analysis (RDA) of the sedimentary communities at genus level. At each step the variable giving the largest drop in Akaike Information Criterion (AIC) and a permutation $p < 0.05$ was added to the model; subsequent steps tested the remaining candidates. Final model: NO_3^- + Fe(III). Predictors were pre-filtered for multicollinearity ($|r| < 0.70$; VIF ≤ 10).

Start: ~ Null model				
**Variable**	**Df**	**AIC**	**F**	**Pr(>F)**
NO_3^-	1	-31.06	5.0747	**0.01***
Fe(III)	1	-28.85	2.5393	**0.045***
Sand	1	-28.087	1.7671	0.105
NO_2^-	1	-27.976	1.6583	0.155
TN	1	-26.768	0.5424	0.795
NH_4^+	1	-26.426	0.246	0.96
Step 1: ~ NO_3^-				
**Variable**	**Df**	**AIC**	**F**	**Pr(>F)**
Fe (III)	1	-32.536	3.0243	**0.01***
NO_2^-	1	-30.06	0.7823	0.495
Sand	1	-30.091	0.8075	0.645
TN	1	-29.785	0.5604	0.805
NH_4^+	1	-29.514	0.3474	0.935
Step 2: ~ NO_3^- + Fe (III)				
**Variable**	**Df**	**AIC**	**F**	**Pr(>F)**
TN	1	-32.617	1.515	0.155
Sand	1	-32.48	1.4069	0.205
NO_2^-	1	-31.968	1.014	0.415
NH_4^+	1	-31.423	0.6137	0.76

* $p < 0.05$.

Notes:

a. Highly correlated variable pairs (Pearson $|r| \geq 0.70$) were as follows:
   - pH with Sand and Silt ($|r| = 0.703$ each)
   - Silt with Sand ($|r| = 1.00$), TOC ($|r| = 0.821$), and TN ($|r| = 0.787$)
   - TOC with Sand ($|r| = 0.821$), Silt ($|r| = 0.820$), and TN ($|r| = 0.976$).
b. The variable Fe(II) had VIF criteria = 10.476.

Table S5. Summary statistics of the RDA final model between the sedimentary communities at genus level and the significant environmental variables (NO_3^- and Fe(III)).

**Summary RDA**			
Component		*Value*	
Constrained variance		0.04458	
Proportion of variance explained (constrained)		50.35%	
Unconstrained variance		0.04397	
Proportion of variance explained (unconstrained)		49.65%	
R^2		0.5035	
Adjusted R^2		0.3931	
**Variance explained by constrained axes**			
Axis	*Eigenvalue*	*% of total variance*	*% of constrained variance*
RDA1	0.0299	44.09%	59.88%
RDA2	0.01468	24.28%	40.12%
**Permutation tests (999 permutations)**			
Test type	*F value*	*p-value*	*Significance*
Global model	4.5631	0.002	**Yes**
Axis 1 (RDA1)	—	0.002	**Yes**
Axis 2 (RDA2)	—	0.009	**Yes**
NO_3^-	—	0.001	**Yes**
Fe(III)	—	0.017	**Yes**

Table S6. Stepwise permutation test (ordistep function, 999 permutations) used to select environmental predictors for the RDA of predicted functional gene counts. At each step the variable giving the largest drop in AIC and a permutation $p < 0.05$ was added to the model; subsequent steps tested the remaining candidates. Final model: NO_3^- + Fe(III). Predictors were pre-filtered for multicollinearity ($|r| < 0.70$; VIF ≤ 10).

Start: ~ Null model				
**Variable**	**Df**	**AIC**	**F**	**Pr(>F)**
NO_3^-	1	-35.352	2.7064	0.005*
Fe(III)	1	-34.574	1.9085	0.045*
Sand	1	-34.296	1.6353	0.075
NO_2^-	1	-33.608	0.9877	0.435
TN	1	-33.478	0.8691	0.595
NH_4^+	1	-33.157	0.5823	0.88
Step 1: ~ NO_3^-				
**Variable**	**Df**	**AIC**	**F**	**Pr(>F)**
Fe (III)	1	-35.78	2.0181	0.01*
Sand	1	-34.787	1.1427	0.325
TN	1	-34.532	0.9298	0.535
NO_2^-	1	-34.301	0.7402	0.61
NH_4^+	1	-34.244	0.6945	0.79
Step 2: ~ NO_3^- + Fe (III)				
**Variable**	**Df**	**AIC**	**F**	**Pr(>F)**
Sand	1	-35.866	1.5188	0.06
TN	1	-35.915	1.5576	0.09
NO_2^-	1	-34.984	0.8444	0.47
NH_4^+	1	-34.916	0.7945	0.725

* $p < 0.05$

Notes:

c. Highly correlated variable pairs (Pearson $|r| \geq 0.70$) were as follows:
   - pH with Sand and Silt ($|r| = 0.703$ each)
   - Silt with Sand ($|r| = 1.00$), TOC ($|r| = 0.821$), and TN ($|r| = 0.787$)
   - TOC with Sand ($|r| = 0.821$), Silt ($|r| = 0.820$), and TN ($|r| = 0.976$).

d. The variable Fe(II) had VIF criteria = 10.476.

Table S7. Summary statistics of the RDA final model between the predicted functional gene counts and the significant environmental variables (NO_3^- and Fe(III)).

**Summary RDA**			
Component		*Value*	
Constrained variance		0.01864	
Proportion of variance explained (constrained)		35.71%	
Unconstrained variance		0.03355	
Proportion of variance explained (unconstrained)		64.29%	
R^2		0.3571	
Adjusted R^2		0.2143	
**Variance explained by constrained axes**			
Axis	*Eigenvalue*	*% of total variance*	*% of constrained variance*
RDA1	0.01116	24.93%	67.08%
RDA2	0.00748	20.19%	32.93%
**Permutation tests (999 permutations)**			
Test type	*F value*	*p-value*	*Significance*
Global model	2.5000	0.002	**Yes**
Axis 1 (RDA1)	—	0.003	**Yes**
Axis 2 (RDA2)	—	0.025	**Yes**
NO_3^-	—	0.002	**Yes**
Fe(III)	—	0.020	**Yes**
